# Study of Winding Short Circuit Characteristics Under Different Insulation Material Temperatures in Transformers

**DOI:** 10.3390/ma18235273

**Published:** 2025-11-21

**Authors:** Xiu Zhou, Yukun Ma, Xiaokang Wang, Tian Tian, Chenfan Tai, Dezhi Chen, Sijun Wang

**Affiliations:** 1 State Grid Ningxia Electric Power Co., Ltd., Electric Power Research Institute, Yinchuan 750011, China; 2 State Grid Ningxia Electric Power Co., Ltd., Wuzhong Power Supply Company, Wuzhong 751100, China; 3 Key Laboratory of Special Motors and High Voltage Electrical Appliances, Ministry of Education, Shenyang University of Technology, Shenyang 110870, China

**Keywords:** transformer, insulation component temperature, material properties, short-circuit resistance, mechanical calculation, transformer component temperature, random forest algorithm

## Abstract

**Highlights:**

**What are the main findings?**

- First bullet: For conductors, the elastic modulus (from 152 MPa at 25 °C to 120 MPa at 120 °C, decreasing by 20%) and tensile strength (from 350 MPa at 25 °C to 273 MPa at 120 °C) decrease with increasing temperature, while plasticity weakens (fracture strain drops 12%); for insulating blocks, the elastic modulus shows accelerated attenuation (from 173.29 MPa at 25 °C to 157.14 MPa at 120 °C) with temperature, and displacement increases (from 0.6–1.82 mm at 25 °C to 1.5–5.5 mm at 120 °C). Meanwhile, higher temperatures lead to increased winding magnetic field intensity (0.339 T to 0.482 T from 25 °C to 120 °C), axial/radial electromagnetic forces, and end displacement (0.903 mm to 1.5 mm from 25 °C to 120 °C).
- Second bullet: The Random Forest algorithm achieves a high-precision prediction of transformer winding short-circuit displacement. The model has a coefficient of determination (R^2^) of 0.9837 and a small root mean square error (RMSE = 0.031280), which can accurately capture the positive correlation between displacement and both operating temperature and number of short-circuit impacts, and can establish a reliable displacement prediction model.

**What is the implication of the main finding?**

- First bullet: For insulating blocks, it fills the gap in previous studies that ignored temperature effects on winding material properties and short-circuit performance. The proposed calculation method for the transformer winding short-circuit force, considering thermal accumulation, gives rise to a refined analysis of heat transfer and mechanics, making the evaluation of the transformer short-circuit withstand capability more accurate (especially under thermal operating conditions) and providing a theoretical basis for optimizing transformer design and manufacturing.
- Second bullet: The high-precision Random Forest prediction model provides a new practical tool for power system operation and maintenance. It can quickly predict winding displacement under different thermal accumulation and impact times, helping to monitor the health status of transformers in real time, assess potential risks of insulation damage or structural deformation, and further ensure the safe and stable operation of power grids.

**Abstract:**

The short-circuit tolerance capability of a transformer is a key performance indicator for ensuring the safe and stable operation of the power system. As the core component of the transformer, the mechanical stability of the windings under the huge electromagnetic force generated by the short-circuit current directly determines the short-circuit tolerance capability of the transformer. Most current research focuses on the coupling analysis of electromagnetic fields and structural fields, while ignoring the influence of temperature, a crucial variable, on the mechanical properties of the winding materials. Therefore, this study conducted tests on the transformer winding conductors, insulating materials, and silicon steel sheet materials under different temperatures, and provided a mathematical model and variation rules of elastic modulus with temperature and the B-H curves of silicon steel sheets at different temperatures. Based on this, a calculation method considering the short-circuit force of the transformer winding under different temperatures of the transformer components was proposed. This method enables precise calculations of the transformer’s mechanics under different temperatures and shows the distribution of leakage magnetic field, short-circuit force, and displacement of the winding under different transformer component temperatures. Finally, the Random Forest algorithm was used to estimate the short-circuit displacement of the transformer winding under different transformer component temperatures, and a short-circuit displacement prediction model based on temperature and impact frequency was provided. This offers a new method for evaluating the short-circuit capacity of the transformer. The feasibility of the calculation method was verified using a 750 kV transformer.

## 1. Introduction

Power transformers, which are key equipment for power transmission and voltage conversion in power grids, have operational reliability that is directly related to the safety and stability of the entire power system. In recent years, with the continuous expansion of the power grid scale and the steady increase in short-circuit capacity, the risk of transformers suffering from short-circuit impacts during operation has increased accordingly. When a short-circuit fault occurs, the enormous short-circuit current generates extremely strong electrodynamic forces in the transformer windings, which act violently within an extremely short time (usually measured in milliseconds). This may cause permanent damage such as winding distortion, collapse, and insulation damage, leading to huge economic losses and power outage accidents. Therefore, the short-circuit withstand capability of transformer windings has always been a core concern for the design, manufacturing, and operation departments of power equipment.

For a long time, scholars and engineers at home and abroad have conducted extensive research on the short-circuit withstand capability of transformer windings, focusing primarily on aspects such as the accurate calculation of short-circuit electromagnetic forces, optimization of the mechanical strength of winding structures, analysis of dynamic response processes, and short-circuit test technologies. Most of these studies are based on the implicit assumption that the mechanical property parameters of the winding materials (such as elastic modulus and yield strength) are constant. However, during the actual operation of a transformer, its windings generate heat owing to load changes, and the operating temperature usually fluctuates between several tens of degrees and above 100 °C. Mechanics of materials has long shown that the mechanical properties of metallic materials (e.g., winding copper conductors) and polymer insulating materials (e.g., insulating blocks and paperboards) are highly temperature sensitive. Changes in temperature can significantly affect the stiffness, strength, and deformation behavior of materials. Therefore, neglecting the temperature effect may lead to deviations in the evaluation of the mechanical state of the windings, failing to truly reflect the short-circuit withstand capability of transformers, especially under thermal operating conditions.

The severe impact of short-circuit current mainly causes two types of hazards. First, a sudden large current causes the winding temperature to rise to quickly exceed the safety limit, resulting in thermal damage or even failure of insulating materials [1,2]; Li Changyun et al. conducted accelerated mechanical-thermal aging tests on insulating paper at 130 °C under four stress levels and three stress frequencies, to study the laws of the mechanical properties of insulating paper under the synergistic effect of mechanical and thermal factors [3]. Fan et al. studied the changes in the mechanical properties of insulating paperboards during thermal aging through accelerated thermal aging tests at 120 °C, and obtained the law that the stress–strain curve of insulating paperboards shifts gradually with the deepening of the aging degree; that is, the strain of the paperboard increases gradually under the same stress, and thermal aging enhances the plastic deformation of the paperboard [4]. BAKSHI reported that the cumulative effect of short-circuit forces has a non-negligible impact on the relevant mechanical characteristics of windings [5]. Temperature changes can significantly alter the constitutive relation of conductor materials, leading to nonlinear differences in mechanical responses such as tensile strength and elongation, even for conductors with the same cross-sectional area when subjected to short-circuit electrodynamic forces of the same amplitude, which directly affect the dynamic stability of transformer windings during the short-circuit transient process [6]. Sinha and Kaur calculated the leakage magnetic field distribution of each wire segment and the short-circuit electromagnetic forces acting on the wire segments when a 630 kVA distribution transformer experienced a three-phase symmetrical short-circuit fault using the finite element method (FEM) [7]. Ahn H M, Lee J Y, and Kim J K established a three-dimensional simulation model of a transformer using the finite element method and conducted a simulation analysis on the magnetic field distribution laws and winding force conditions before and after the transformer’s short-circuit fault [8]. Bakshi A, Kulkarni S V, and Bakshi A pointed out that the torsional deformation of helical windings is caused by the combined action of axial current components and radial leakage magnetic fields, which considered the influence of the transformer structure, material properties, and other factors on the torsional electromagnetic forces induced by axial currents and determined the circumferential displacement of helical windings [9]. Liu Jiaji et al. established a two-dimensional equivalent model for a 110 kV three-phase double-winding transformer using COMSOL 6.3 version software, and presented the distribution of radial and axial leakage magnetic fields in the wire segments at the ends and middle of the high-voltage and low-voltage windings [10]. Bakshi studied the influence of strain on the mechanical strength of transformer windings under short-circuit conditions. He investigated the distribution of residual strain in windings during the short-circuit process using an analytical method and calculated the strain generated in the windings under the action of radial short-circuit forces. Considering the existence of residual strain in conductors, he determined the mechanical strength of a transformer under short-circuit conditions [5]. Yadav studied transformer electromagnetic forces and winding deformations caused by short-circuit currents. Axial and radial forces are generated in transformer windings, leading to their deformation [11]. Luo Hanwu et al. used a 110 kV power transformer as an example, obtained the variation law of the elastic modulus of transformer insulating blocks with temperature using a dynamic thermomechanical analyzer, and conducted a theoretical analysis on the variation law of the elastic modulus of copper conductors with temperature [12]. Muhamad et al. analyzed the temperature changes inside and outside the transformer tank by studying the influence of the hot-spot temperature on the overall temperature distribution of the transformer and the thermal distribution related to the hot-spot temperature [13]. Bo calculated the temperature field of a power transformer using the finite element simulation software Fluent by establishing a three-dimensional model of the transformer, obtained the temperature distribution law of each winding of the transformer, and studied the short-circuit thermal stability of the windings [14]. Aboura and Touhami studied winding materials and found that materials with good electrical conductivity, such as copper or aluminum, exhibit good performance under normal operating conditions but may be subjected to extremely high current impacts under short-circuit conditions [15]. Brown et al. proposed a new winding design method and a thermal stability improvement scheme by optimizing the winding structure, improving the material thermal stability, and enhancing the winding cooling system [16]. Yongteng et al. established an electromagnetic–temperature field simulation platform for a new type of transformer, conducted a temperature field simulation on it, and experimentally verified the correctness of the proposed scheme [17]. Li Zhongxiang et al. conducted an inspection and analysis on a short-circuit fault accident of a 220 kV transformer; by rechecking the short-circuit strength of the transformer, they concluded that the failure of the self-adhesive effect of the transformer’s transposed conductors under the action of long-term thermal effects was the main cause of this short-circuit fault accident [18]. Wang Junyang analyzed the temperature, oil flow characteristics, and short-circuit electromagnetic characteristics of power transformers [19]. Jianbin et al. proposed a novel multi-fault diagnosis method based on a Random Forest algorithm. This approach extracts fault features via wavelet packet decomposition, selects features using the ReliefF algorithm, and constructs a Random Forest classifier for fault identification [20]. Li et al. introduced a transformer fault detection technique utilizing an improved Random Forest algorithm [21]. Wang Lei et al. developed a method for monitoring the operational status of equipment in smart substations based on the Random Forest algorithm [22]. Luo Yong established an early warning method for abnormal states in 220 kV main transformers, building upon the Random Forest method, which plays a significant role in ensuring secure and stable operation of the power system [23]. Long et al. presented a method for multi-source monitoring of transformer temperature and fault prediction by grating fog computing with the Random Forest algorithm [24].

Despite the progress made in existing studies, three critical gaps remain unresolved, which limit the accuracy of transformer short-circuit withstand capability evaluation and real-time maintenance support: Luo et al. [12] and Li et al. [3]) have explored the temperature influence on single material parameters (e.g., elastic modulus of insulating blocks) but failed to establish a comprehensive mathematical model for the temperature dependence of key mechanical parameters (elastic modulus, tensile strength, yield strength, and fracture strain) of both winding conductors (copper) and insulating materials (polymer blocks). This lack of systematic parameter modeling makes it impossible to accurately input temperature-sensitive material data into electromagnetic–mechanical coupling simulations. Circuit force analysis (e.g., Ahn et al. [8] and Sinha et al. [7]) focuses on electromagnetic–structural field coupling but ignores the cumulative effect of long-term thermal operation on material properties and winding mechanical response. No existing method integrates thermal accumulation into short-circuit force calculation, leading to deviations in evaluating winding displacement and stress under actual long-term thermal operating conditions (e.g., 80–120 °C). Long et al. [24] and Luo et al. [23] use Random Forest or similar algorithms for transformer fault prediction, but rarely couple two key factors—operating temperature and number of short-circuit impacts—to predict winding short-circuit displacement. This results in low prediction accuracy for displacement under varying thermal accumulation and impact frequency, failing to support real-time health monitoring of transformers.

To address the above gaps, this study focuses on 110 kV power transformers and sets the following research objectives. (1) Static mechanical tests on transformer winding conductors (copper) and insulating blocks use an HSD-LX304B high–low temperature universal testing machine (20–120 °C). Quantify the variation laws of elastic modulus, tensile strength, yield strength, and displacement with temperature, and establish polynomial fitting models (for conductors and insulators) to provide accurate material input parameters for subsequent simulations. (2) Establish temperature-dependent material models in a 3D finite element model of a 110 kV transformer (50,000 kVA). Calculate and analyze the distribution characteristics of winding leakage magnetic field, axial/radial electromagnetic force, and displacement under different thermal accumulation conditions (25–120 °C) to analyze the refined coupling of thermal–magnetic–mechanical fields. (3) Use temperature and a number of short-circuit impacts as input features and winding displacement as the output. Verify the model’s accuracy using test and simulation data (target: coefficient of determination R^2^ ≥ 0.98, with a root mean squared error RMSE ≤ 0.04), to provide a practical tool for evaluating transformer short-circuit withstand capability and real-time maintenance.

Existing studies have conducted multi-dimensional research on transformer winding short-circuit withstand capacity, achieving results in aspects such as accurate calculation of short-circuit electromagnetic forces, optimization of the mechanical strength of winding structures, research on the thermal aging characteristics of insulating materials, and application of algorithms such as Random Forest in fault diagnosis, but there are still three key limitations: although some studies have focused on the temperature sensitivity of mechanical parameters of a single material, no systematic temperature-dependent mathematical models have been established for the full-dimensional key mechanical parameters of winding conductors and insulating materials, making it difficult to accurately input temperature-sensitive material parameters into electromagnetic–mechanical coupling simulations; most existing short-circuit force analyses focus on electromagnetic–structural field coupling, ignoring the cumulative effect of long-term thermal operation on material properties and winding mechanical responses, and lacking effective methods to incorporate thermal accumulation into short-circuit force calculations, resulting in deviations in the evaluation of winding displacement and stress under actual thermal operating conditions; and transformer fault prediction research based on Random Forest mostly focuses on temperature monitoring or fault type identification, and couples operating temperature and number of short-circuit impacts to predict winding short-circuit displacement, whose prediction accuracy is difficult to meet real-time operation and maintenance requirements. To address these research gaps, this study takes a 110 kV/50,000 kVA power transformer as the research object, aiming to establish high-precision polynomial fitting models for temperature-sensitive parameters through static mechanical tests of winding conductors and insulation blocks in the temperature range of 25–120 °C, achieve refined magnetic-force field coupling analysis by embedding temperature-dependent material models into three-dimensional finite element models, and construct high-precision Random Forest prediction models for winding short-circuit displacement with operating temperature and number of short-circuit impacts as inputs, thereby improving the evaluation system of transformer short-circuit withstand capacity.

## 2. Analysis of Mechanical Properties of Winding Conductors and Insulating Materials

The transformer windings are mainly composed of insulating paperboards, flat copper coils, and other fixing components. When a short circuit occurs, the current flowing through the transformer generates enormous short-circuit forces, causing conductor deformation. As an important component of the transformer body, insulating blocks play an important role in stabilizing the transformer structure, ensuring the insulation distance between the coils, and improving the operational stability of the transformer. This chapter uses a high–low temperature universal testing machine to simulate the compaction process of transformer windings, study the compression process of insulating blocks under static force and cyclic dynamic force loads at different temperatures, measure the material parameter characteristic values of insulating blocks, and provide reliable data for establishing the finite element model of transformer windings.

### 2.1. Test Instruments and Specimens

**Figure 1:**
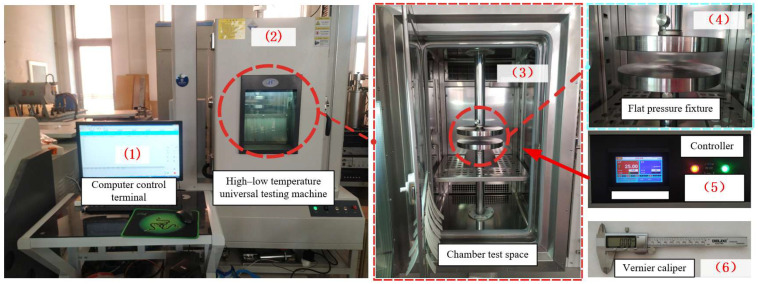
Schematic diagram of the compression test device.

The test platform specifically constructed for conducting compression performance tests is schematically depicted in Figure 1. This platform integrates multiple functional modules to ensure the accuracy, stability, and controllability of the compression test process. The following are among the core components of the platform: (1) represents the computer control terminal, which serves as the central command and data processing hub. It is responsible for issuing pre-set test control parameters (e.g., load rate, target temperature, holding time), real-time acquisition and visualization of test data (e.g., compressive load, displacement, environmental temperature), and automatic storage of test records for subsequent data analysis. (2) refers to the high–low temperature universal testing machine, the core load-applying and environmental control device of the platform. This machine is capable of providing a continuous and stable compressive load within a specified range (in line with test standards) and regulating the internal environmental temperature between −70 °C and 150 °C (or other customized ranges), thereby simulating the compression behavior of specimens under different thermal conditions. (3) denotes the chamber test space, an enclosed sub-module integrated within the high–low temperature universal testing machine. It is designed to maintain the target temperature field generated by the machine’s temperature control system, ensuring that the test specimen is in a uniform and stable thermal environment throughout the compression process, and avoiding temperature fluctuations that may affect test results. (4) stands for the flat pressure fixture, a specialized clamping and force-transmission component. Its upper and lower surfaces are precision-machined to ensure high flatness (≤0.01 mm/m) and parallelism, which enables the uniform transmission of the compressive load from the testing machine to the two end faces of the specimen, and effectively prevents eccentric loading (a key factor leading to test result deviations) during the compression process. (5) indicates the controller, an intermediate control unit that establishes communication between the computer control terminal and the high–low temperature universal testing machine. It converts the digital control commands from the terminal into analog signals recognizable by the testing machine (e.g., adjusting the load actuator’s movement speed, activating the temperature control system) and feeds back the real-time operating status of the testing machine (e.g., load output accuracy, temperature control deviation) to the terminal for dynamic adjustment. (6) refers to the vernier caliper (with a measurement accuracy of 0.02 mm), a pre-test and post-test dimensional measurement tool. It is used to measure the key geometric parameters of the specimen before the test (e.g., cross-sectional area and height) and the residual deformation (e.g., lateral expansion and axial shortening) of the specimen after the test, providing essential geometric data for calculating compression performance indices such as compressive strength (σ = F/A, where F is the maximum compressive load and A is the initial cross-sectional area) and compressive modulus.

The HSD-LX304B high–low temperature universal testing machine was used for the test. The testing machine features simple operation and ultra-high testing precision, with a force measurement accuracy better than grade 0.5, a force resolution of 1/200,000, a deformation measurement resolution up to 0.0001 mm, a maximum capacity of 5 tons, and a diameter of 100 mm for the upper and lower fixture disks, which can meet the test requirements. This test was conducted under different temperature conditions (20, 40, 60, 80, 100, and 120) to perform mechanical tests on transformer insulating pads and conductor blocks. To reduce the test errors, three groups of measurements were performed on the same-type pads and copper conductor rods. The specific test materials used were as follows.

**Figure 2:**
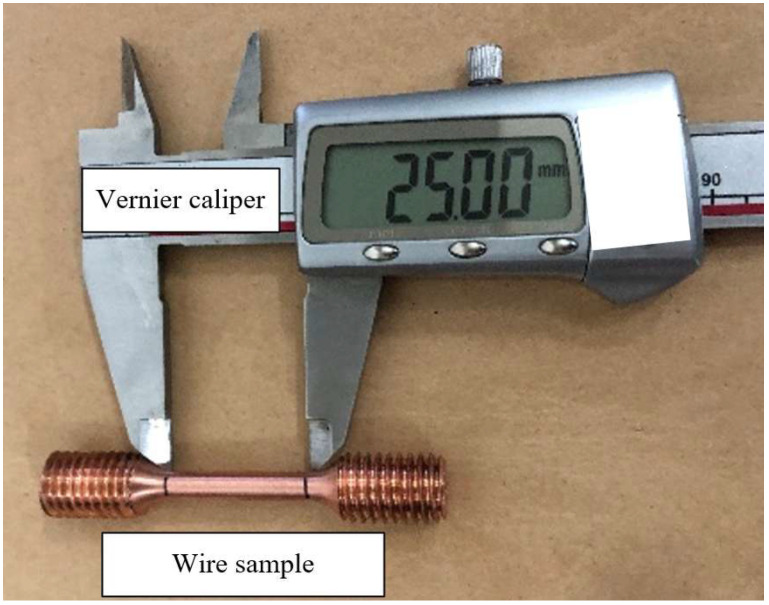
Copper conductor tensile specimen rod.

To test the key characteristic parameters of transformer conductors under different temperature conditions, as shown in Figure 2, transformer conductor specimens were first prepared, and the test equipment was adjusted to the state conforming to national standard testing. Marked reference lines on the parallel middle part of the specimen to indicate the gauge length; these reference lines have no impact on the test results. The width and thickness of the parallel middle part of the specimen were accurate to 0.01 mm. Five points were measured for each specimen, and the average value was obtained using the median-mean filtering method.

**Figure 3:**
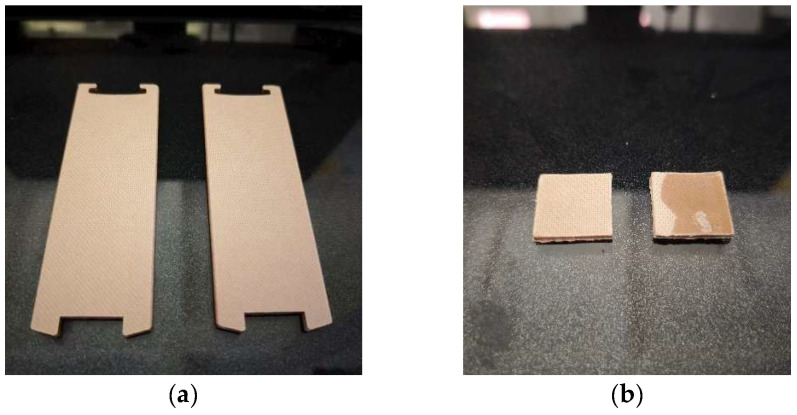
Schematic diagram of insulating blocks. (**a**) Transformer insulators; (**b**) Transformer insulation after cutting).

The transformer insulation blocks are shown in Figure 3a. To ensure that the test equipment can exert sufficient pressure on the transformer insulating blocks, various blocks were reprocessed before the test to make square specimens with a standard width of 20 mm, and the thickness of the specimens was the same as that of the blocks. The reprocessed block specimens are shown in Figure 3b.

### 2.2. Analysis of the Tensile Mechanical Properties of the Winding Conductors

**Figure 4:**
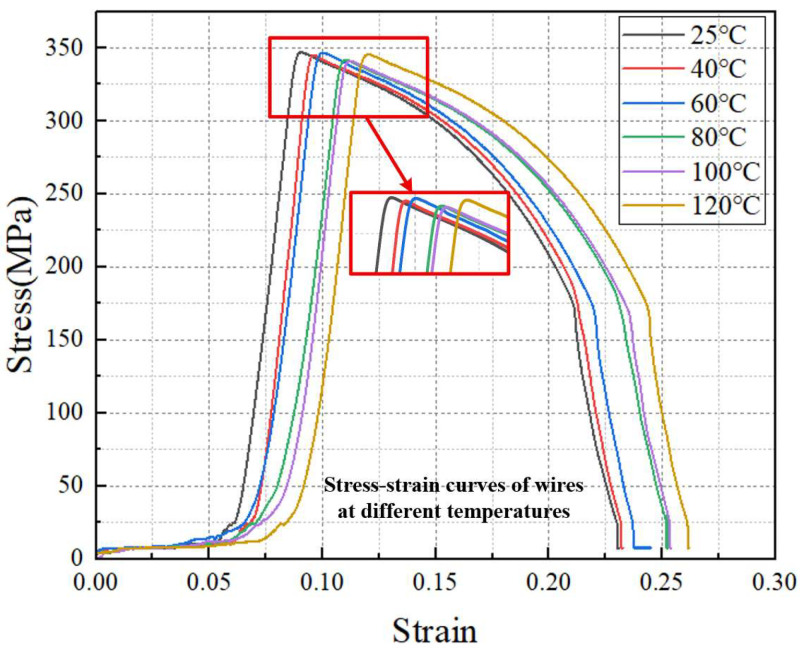
Stress–strain curves of transformer conductors at different temperatures.

The mechanical property test results of transformer copper conductors under different temperatures are presented in Figure 4. To ensure the comparability of test results, homogeneous specimens of the same type were selected for testing—all specimens were subject to strict pre-test screening. Dimensional deviation was controlled within ±0.05 mm, and surface defects were excluded to avoid interference with stress–strain response.

Prior to mechanical property measurement, the selected conductor specimens were placed in the chamber test space of the HSD-LX304B high–low temperature universal testing machine and subjected to a programmed temperature control process: heated at a rate of 5 °C/min until the target test temperatures are reached (20, 40, 60, 80, 100, 120 °C, consistent with Section 2), then held at each temperature for 30 min to ensure the specimen interior reached thermal equilibrium.

Subsequently, mechanical compression tests were performed on the thermally equilibrated specimens at a constant load rate of 2 mm/min. During the test, the machine’s data acquisition system recorded compressive stress and axial strain at a 10 Hz sampling frequency. Based on these data, the stress–strain curves of transformer copper conductors at each target temperature were generated, which intuitively reflect the variation in the conductor’s elastic deformation, yield behavior, and plastic deformation stages with temperature.

It can be seen from the measurement results that the temperature has a significant impact on the mechanical properties of transformer conductors. In the elastic stage, the elastic modulus of the transformer conductor was 152 GPa at 25 °C and 120 GPa at 120 °C, and the elastic modulus decreased by 20% as the temperature increased, which directly reflects the weakening of the material’s elastic properties caused by elevated temperatures. In the yielding and strengthening stages, the stress peaks of the transformer conductor were 350, 341 GPa at 40 °C, 328 GPa at 60 °C, 309 GPa at 80 °C, 292 GPa at 100 °C, and 273 GPa at 25, 40, 60, 80, 100, and 120 °C, respectively. From the numerical changes, it can be observed that the tensile strength exhibits a relatively evident decrease with each increase in temperature, and the rate of decrease tends to accelerate as the temperature increases. For example, the tensile strength decreased by 5.7% from 25 °C to 60 °C, whereas it decreased by 12.9% from 80 °C to 120 °C, reflecting the adverse effect of high temperature on the yielding and strengthening properties of the material. In the necking and fracture stage, the fracture strain of the transformer conductor is 0.25 at 25 °C and 0.22 at 120 °C, with a 12% decrease in fracture strain, indicating a reduction in the material’s plastic deformation capacity and an increase in brittleness at high temperatures.

In the elastic deformation stage, as the temperature increases, the elastic modulus of the conductor exhibits a decreasing trend, meaning that the material’s ability to resist elastic deformation weakens; entering the yielding and strengthening stage, the higher the temperature, the more significantly the yield strength and tensile strength of the conductor decrease, indicating a substantial reduction in the load-bearing capacity of the material at high temperatures. At the necking and fracture stage, high temperature weakens the conductor’s plastic deformation capacity, making it more prone to brittle fracture and resulting in poorer plasticity.

**Figure 5:**
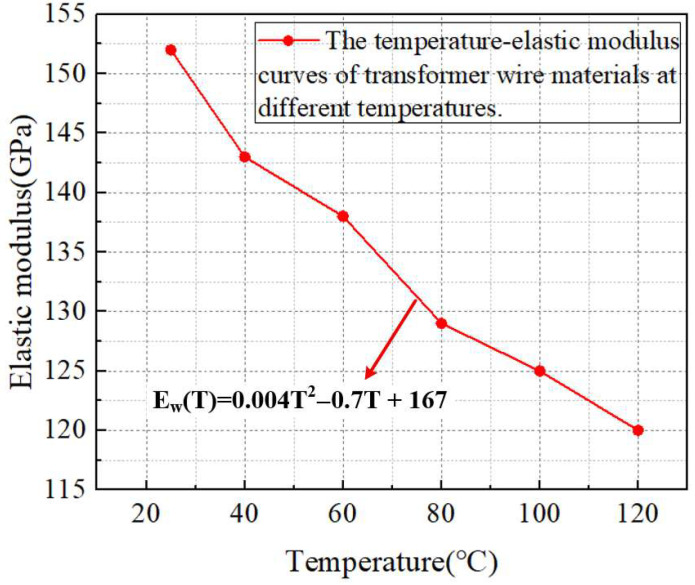
The temperature–elastic modulus curves of transformer wire materials at different temperatures.

The temperature–elastic modulus test results of transformer wire materials are shown in Figure 5.

Through polynomial fitting, curve fitting is conducted on the temperature–elastic modulus of transformer winding materials, and Formula (1) is obtained:

(1) $$E_{w}(T)=0.004T^{2}-0.7T\;+\;167$$

Herein, E_w_ represents the elastic modulus of the winding material at different temperatures (GPa), and T denotes the temperature endured by the winding material (°C). As can be seen from Formula (1), in terms of the variation relationship between temperature and elastic modulus, when the temperature is 25 °C, the elastic modulus of the transformer wire material is approximately 152 GPa; as the temperature rises to 40 °C, the elastic modulus decreases to about 143 GPa; when the temperature further increases to 60 °C, the elastic modulus drops to approximately 138 GPa; when the temperature reaches 80 °C, the elastic modulus is about 129 GPa; at 100 °C, the elastic modulus decreases to around 125 GPa; and when the temperature rises to 120 °C, the elastic modulus reduces to about 120 GPa. Within the temperature range of 25 °C to 120 °C, the elastic modulus of the transformer wire material exhibits a continuous and relatively significant decreasing trend with the increase in temperature. Furthermore, the decline range of the elastic modulus is relatively larger in the early stage, and although the decline rate slows down in the later stage, it still maintains a stable decreasing trend, clearly demonstrating the weakening effect of temperature rise on the elastic modulus of this material.

**Figure 6:**
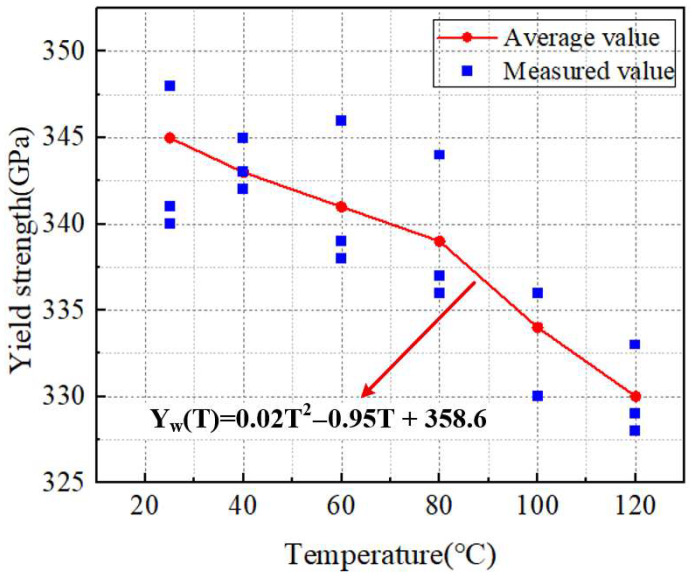
The temperature–strength curves of transformer wire materials at different temperatures.

The test results of Figure 6 were subjected to polynomial fitting, and a curve fitting was conducted on the relationship between the temperature of the transformer winding material and the yield strength, thereby obtaining Formula (2).

(2) $$Y_{w}(T)=0.02T^{2}-0.95T+358.6$$

Herein, Y_w_ denotes the yield strength of the winding material at different temperatures (GPa), and T represents the temperature applied to the winding material (°C). As can be seen from Formula (2), as the temperature of the transformer wire material increases, its yield strength decreases gradually. When the temperature is 25 °C, the yield strength of the transformer wire material is 345 GPa, with the measured values distributed in the range of 340 GPa to 348 GPa; as the temperature rises to 40 °C, the yield strength is 343 GPa, and the measured values are mostly in the range of 342 GPa to 345 GPa; when the temperature increases to 60 °C, the yield strength is 341 GPa, and the measured values are concentrated in the range of 338 GPa to 346 GPa; when the temperature reaches 80 °C, the yield strength is 339 GPa, with the measured values distributed in the range of 336 GPa to 344 GPa; at 100 °C, the yield strength is 344 GPa, and the measured values are approximately 330 GPa and 336 GPa; when the temperature rises to 120 °C, the yield strength is 330 GPa, and the measured values are concentrated in the range of 328 GPa to 333 GPa. This clearly demonstrates the weakening effect of increasing temperature on the yield strength of the transformer wire.

To verify the reliability of the fitting models, statistical analysis was performed on three sets of parallel test data of elastic modulus and yield strength at each temperature point: The standard deviation of elastic modulus is 1.2–2.5 GPa, with a relative standard deviation (RSD) ≤ 1.7%; The standard deviation of yield strength is 2.3–3.1 GPa, and RSD ≤ 0.9%, indicating good test repeatability. Meanwhile, residual analysis was conducted for the polynomial fitting. The maximum absolute residual of elastic modulus is 0.8 MPa, and that of yield strength is 0.5 MPa; the residuals follow a normal distribution without systematic deviation, demonstrating high accuracy of the fitting models.

### 2.3. Analysis of the Mechanical Compression Performance of Insulating Materials

**Figure 7:**
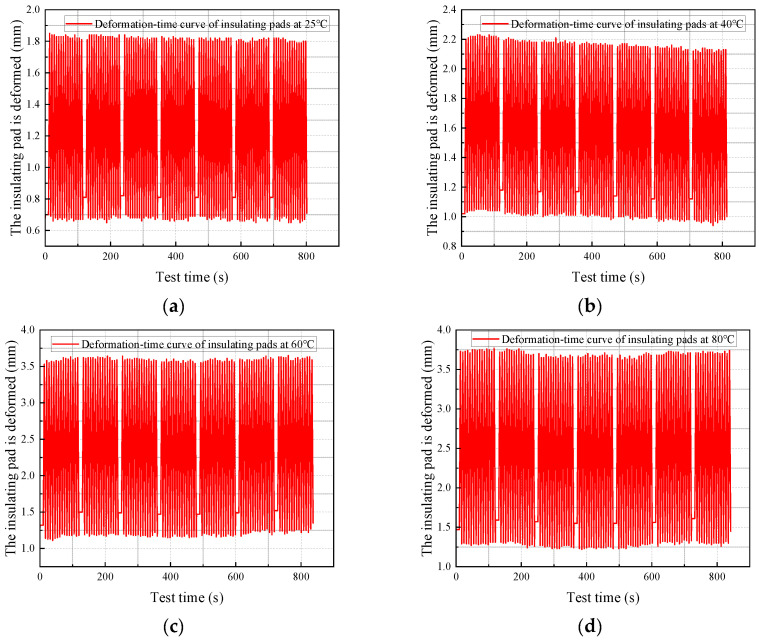

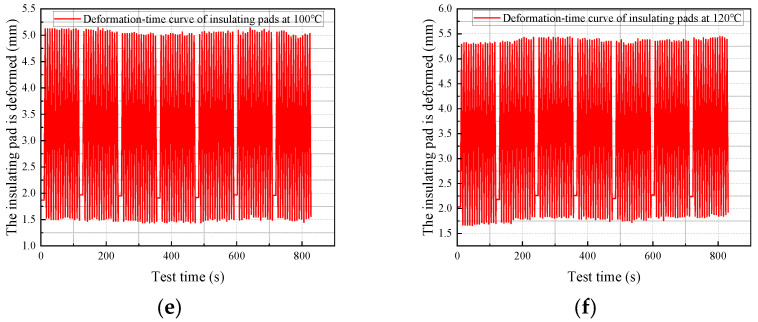
Displacement–time curves of transformer insulating blocks at different temperatures. (**a**) 25 °C, (**b**) 40 °C, (**c**) 60 °C, (**d**) 80 °C, (**e**) 100 °C, (**f**) 120 °C.

The test results for the mechanical properties of the transformer insulating blocks at different temperatures are shown in Figure 7. The test selected same-type insulating blocks for measurement. The insulating block specimens were heated to the test-specified temperatures before measurement, and the time–displacement curves and temperature–elastic modulus curves of the transformer insulating blocks at different temperatures were obtained.

It can be seen from the measurement results that the displacement of the insulating blocks fluctuated within the range of 0.6–1.82 mm at 25 °C. When the temperature rises sequentially to 40 °C, 60 °C, 80 °C, 100 °C, and 120 °C, their displacement ranges expand to 1.25–2.23 mm, 1.27–3.55 mm, 1.5–3.75 mm, 1.5–5.1 mm, and 1.5–5.5 mm, respectively, showing a quantitative growth law of displacement with increasing temperature and revealing the strengthening effect of high temperature on the deformation capacity of the insulating blocks. As a key influencing factor, temperature governs the deformation and mechanical properties of insulating blocks. With an increase in temperature, the displacement of the insulating blocks shows a continuously increasing trend, and the fluctuation amplitude of displacement over time and the degree of dynamic response also increase synchronously, reflecting a decline in the deformation stability of the material at high temperatures.

**Figure 8:**
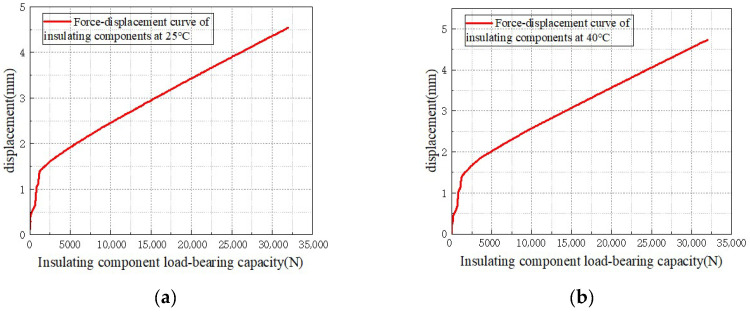

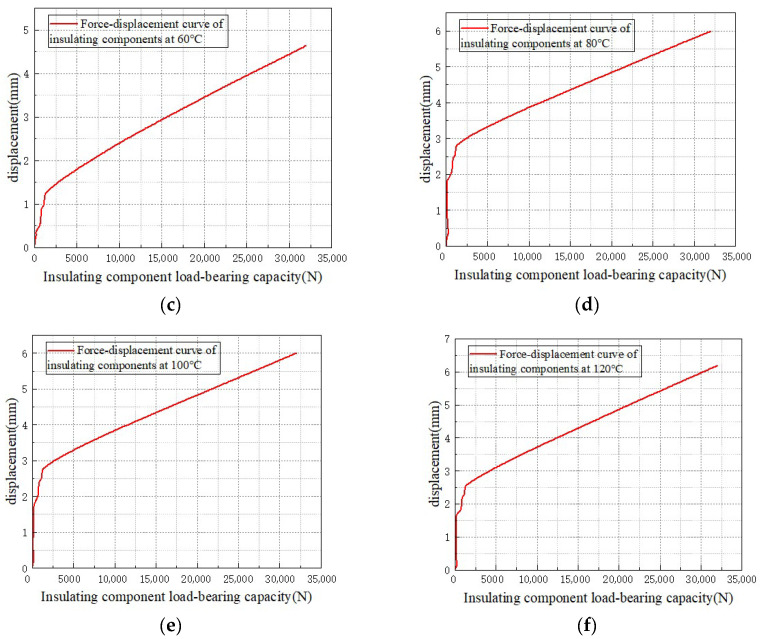
Force–displacement curves of transformer insulation blocks at different temperatures. (**a**) 25 °C, (**b**) 40 °C, (**c**) 60 °C, (**d**) 80 °C, (**e**) 100 °C, (**f**) 120 °C.

As shown in Figure 8, static mechanical experiments were conducted on transformer insulation blocks at different temperatures. Since the maximum allowable value of the cardboard used in the insulation blocks is 80 MPa, the insulation blocks were cut into square pieces of 20 × 20 mm to ensure that the insulation blocks meet the force-bearing standard. A force of 32,000 N was applied, and the force–displacement curves of the transformer insulation blocks at different temperatures were obtained.

Figure 8 shows the force–displacement curves of the transformer insulation pads at different temperatures. It can be observed that in each temperature condition, the curves exhibit the characteristic that the displacement initially increases rapidly with the load, and then enters a relatively stable growth stage. This indicates that the deformation of the insulation pads under load follows an evolutionary pattern of being initially nonlinear and then approximately linear. At the same time, temperature has a significant regulatory effect on its mechanical properties. When the same load is applied, the higher the temperature, the greater the corresponding displacement, suggesting that an increase in temperature will weaken the stiffness characteristics of the insulation pads.

**Figure 9:**
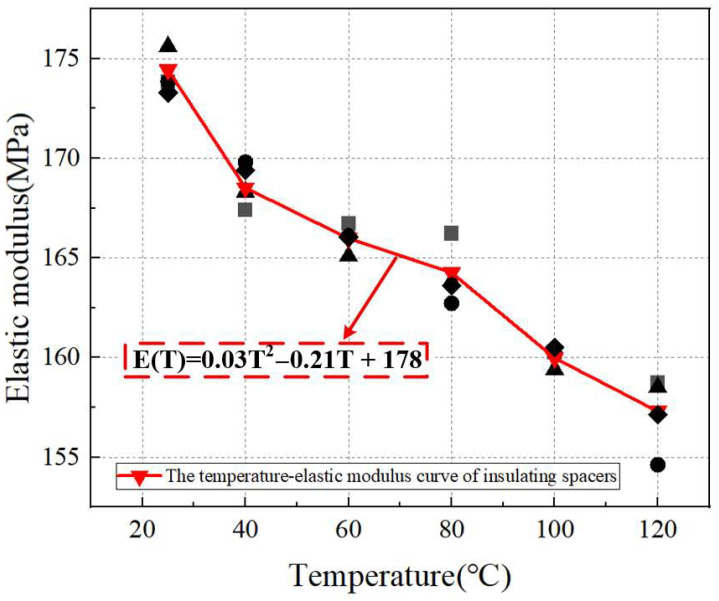
Temperature–elastic modulus curves of transformer insulating blocks at different temperatures.

As shown in Figure 9, static mechanical tests were conducted on the insulating blocks at different temperatures, and the temperature–elastic modulus curve of the transformer insulating blocks was obtained.

By performing polynomial curve fitting on the temperature–elastic modulus curve of the transformer insulating blocks, Formula (3) can be obtained as follows:

(3) $$E(T)=0.03T^{2}-0.21T+178$$

where E is the elastic modulus of the transformer insulating blocks (MPa), and T is the temperature (°C). It can be seen from Equation (1) that the elastic modulus exhibits characteristics of accelerated attenuation as the temperature increases. The quadratic term coefficient is negative and significant, indicating that the higher the temperature, the faster the decline rate of the elastic modulus. When the temperature rises from 25 °C to 120 °C, the elastic modulus decreases from 173.29 MPa to 157.14 MPa, and the amplitude and rate of attenuation intensify as the temperature increases.

For the transformer insulating blocks, the elastic modulus values calculated from the linear elastic segment of stress–strain curves show a monotonic decreasing trend with increasing temperature in the range of 25–120 °C. Based on the average values of three parallel tests, the elastic modulus decreases from 173.29 MPa at 25 °C to 157.14 MPa at 120 °C, with a total reduction of approximately 9.3%.

This monotonic decline directly indicates that temperature is a critical factor weakening the deformation resistance of the insulating blocks. From a micro-mechanical perspective, the epoxy resin matrix in the composite insulating blocks has high glass transition temperature sensitivity: as temperature rises, the thermal motion of epoxy resin molecular chains is enhanced, leading to the weakening of intermolecular van der Waals forces and covalent bond constraints. This reduces the ability of the matrix to transfer stress between reinforcing phases, thereby decreasing the overall elastic modulus of the insulating block and making it more prone to elastic deformation under the same compressive stress.

**Figure 10:**
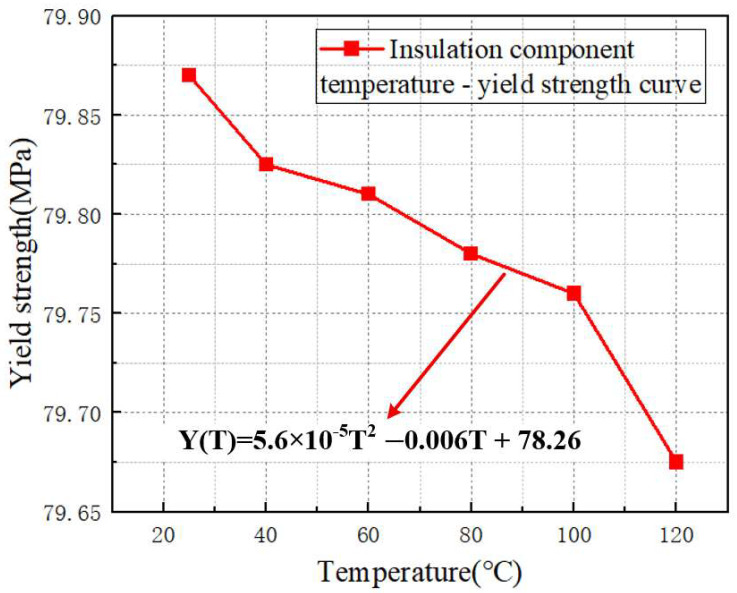
The temperature–yield strength curve of the transformer insulation block under different temperatures.

As shown in Figure 10, static mechanical tests were conducted on the insulating blocks at different temperatures, and the temperature–yield strength curve of the transformer insulating blocks was obtained.

By performing polynomial curve fitting on the temperature–elastic modulus curve of the transformer insulating blocks, Formula (4) can be obtained as follows:

(4) $$Y(T)=0.000056T^{2}-0.006T+78.26$$

where Y is the yield strength of the transformer insulation component (MPa), and T is the temperature (°C). It can be seen from the formula Y(T) = 5.6 × 10^−5^T^2^ − 0.006T + 78.26 that the yield strength exhibits characteristics of continuous attenuation as the temperature increases. The quadratic term coefficient is negative, indicating that the higher the temperature, the faster the decline rate of the yield strength. When the temperature rises from 25 °C to 120 °C, the yield strength decreases from approximately 79.87 MPa to 79.67 MPa, and the amplitude and rate of attenuation become more significant as the temperature increases.

For the transformer insulation component, the yield strength values show a monotonic decreasing trend with increasing temperature in the range of 25–120 °C. Based on the data points, the yield strength decreases from about 79.87 MPa at 25 °C to around 79.67 MPa at 120 °C, with a total reduction of approximately 0.25%.

This monotonic decline directly indicates that temperature is a critical factor weakening the yield resistance of the insulation component. From a micro-mechanical perspective, the insulating material has high temperature sensitivity: as temperature rises, the thermal motion of molecular chains in the insulation material is enhanced, leading to the weakening of intermolecular bonding forces. This reduces the material’s ability to resist plastic deformation initiation, thereby decreasing the overall yield strength of the insulation component and making it more prone to yielding under the same stress.

The statistical results of three sets of parallel test data for the elastic modulus and yield strength of insulation blocks are as follows: the standard deviation of elastic modulus is 0.9–1.8 MPa, with RSD ≤ 1.2%; the standard deviation of yield strength is 0.05–0.08 MPa and RSD ≤ 0.1%, indicating small test discreteness. Fitting residual analysis shows that the maximum residual of elastic modulus is 0.6 MPa, and the maximum residual of yield strength is 0.03 MPa; the residual distribution conforms to random characteristics, and fitting Formulas (3) and (4) can accurately describe the relationship between temperature and mechanical parameters.

### 2.4. Measurement of Magnetic Properties of Silicon Steel Sheets Under Varying Temperatures

**Figure 11:**
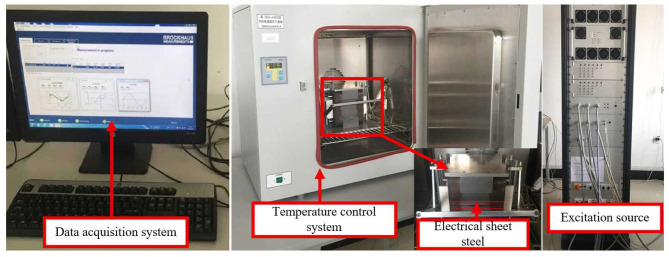
Silicon steel sheet testing system at different temperatures.

Figure 11 shows the test system for silicon steel sheets under varying temperatures. It mainly consists of a temperature control system, a data acquisition system, a silicon steel sheet test device, and an AC-DC hybrid excitation source. The temperature control system ensures the true reflection of the magnetic and mechanical properties of silicon steel sheets during the test by precise temperature control, operating condition simulation, and interference suppression: as the core component, the data acquisition system is responsible for the real-time collection, processing, and analysis of various physical quantities and electrical parameters generated during the test; the AC-DC hybrid excitation source functions to simulate the complex electromagnetic environment in actual operating conditions and comprehensively evaluate the magnetic properties and loss characteristics of silicon steel sheets under different excitation conditions. Table 1 presents the main performance parameters of the test system.

**Figure 12:**
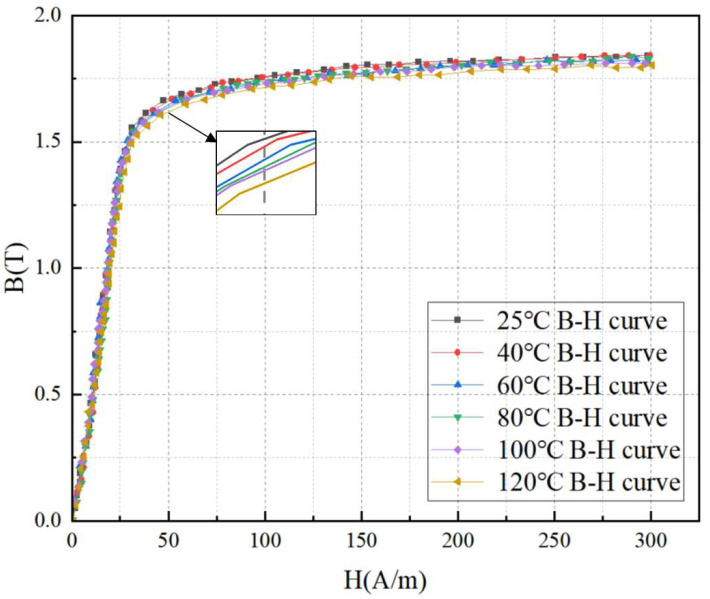
Test results of silicon steel sheets at different temperatures.

Based on the test system shown in Figure 11 and the performance parameters of the test system presented in Table 1, research on the magnetic properties of silicon steel sheets under different conditions can be conducted. The magnetic properties of silicon steel sheets of a 110 kV main transformer were tested at different temperatures, and the B-H curves under varying temperatures were obtained, as shown in Figure 12.

As shown in Figure 12, with the increase in temperature, the linear regions of the core silicon steel sheets overlap and are almost unaffected by temperature, while the B value decreases to a certain extent. This indicates that as the test temperature rises, the magnetic flux density B of the silicon steel sheets gradually decreases, which affects the leakage magnetic flux of the 110 kV transformer and thereby influences its mechanical performance. Therefore, when calculating the mechanical performance parameters of the 110 kV main transformer, the temperature effect on the core silicon steel sheets of the power transformer should be taken into account.

### 2.5. Measurement of Dielectric Constant of Insulation Spacers Under Varying Temperatures

**Figure 13:**
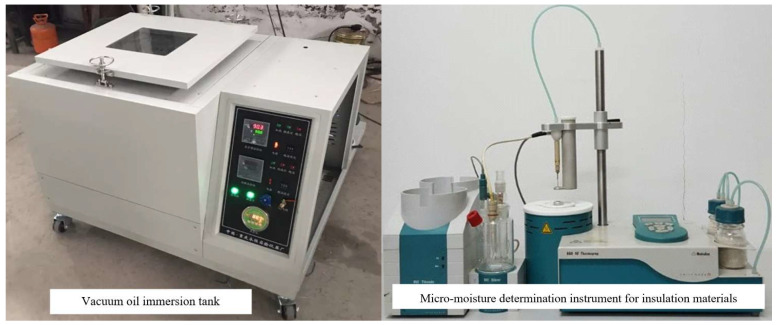
Test preparation instruments for insulation spacer material samples.

The insulation spacer material was cut into circular sheets with a diameter of 20 mm, as shown in Figure 13, to facilitate the measurement of the frequency-domain dielectric spectrum of the insulation paper and the micro-moisture content in the paper. The cut samples were placed in a vacuum oil immersion tank for drying for 24 h, ensuring the initial moisture content was controlled below 0.5%. The vacuum oil immersion tank is shown in Figure 13: Insulation material samples treated as above were taken for moisture content determination, using an 852 KF Moisture Meter produced by Metrohm (Switzerland). The coulometric Karl Fischer moisture meter is suitable for the measurement of micro-moisture content in solids, liquids, and gases, with rapid and accurate measurement performance. It has a detection limit of 1 ppm (especially for measuring moisture content in solvents and gases), with automatic background determination and subtraction, as well as functions of learning titration, online help, and statistical calculation. When determining the moisture content of insoluble or poorly soluble insulation material samples, it is necessary to connect the 860 Thermo Prep Headspace Karl Fischer Sample Heater for accurate measurement; the operating temperature of the drying oven ranges from 50 to 300 °C. The micro-moisture determination equipment for insulation materials is shown in Figure 13.

The dielectric constant of insulation spacers was measured to verify the influence of temperature on their relative dielectric constant. First, the insulation spacer was placed between two gold-plated copper electrodes with a diameter of 4 cm for measurement. The dielectric parameter testing instrument for insulation spacers was the Alpha-A broadband dielectric impedance spectrometer manufactured by Novocontrol GmbH (Germany). This instrument has a frequency range of 3 μHz to 40 MHz, an impedance range of 10 mΩ to 100 TΩ, and a capacitance range of 1 fF to 1 F. Its measurement modes include automatic correction, automatic reference, and manual reference, with a temperature range of −100 to 250 °C. The physical diagram of the Alpha-A broadband dielectric impedance spectrometer is shown in Figure 13. The test measurement frequency range was 10^−2^ to 10^7^ Hz, and the temperature range was −50 to 180 °C. To ensure test repeatability, each test was performed three times. Moreover, to eliminate randomness, the repeated measurements were not conducted on the same sample; instead, three samples were taken from the same state for testing.

**Figure 14:**
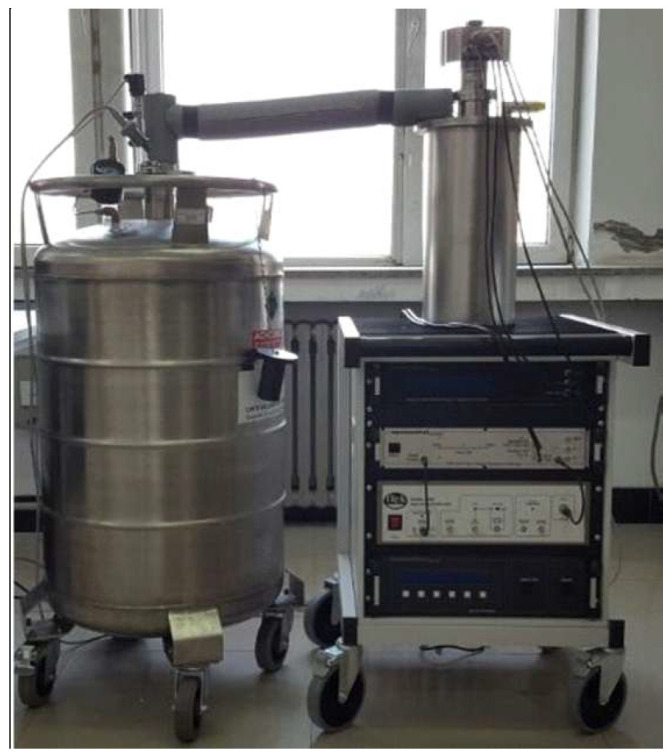
Alpha-A broadband dielectric impedance spectrometer.

The measurement results of the relative dielectric constant of insulation spacers under varying temperatures are shown in Figure 14. The measurement frequency of the insulation spacers was 50 Hz, and the moisture content was maintained at 2%, ensuring that the tested insulation spacer materials are consistent with the actual operating conditions of the transformer.

**Figure 15:**
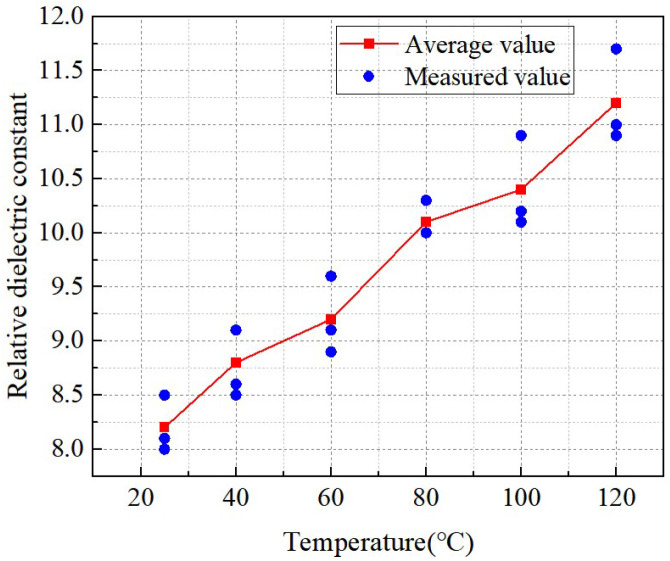
Relative dielectric constant of insulation spacer material under varying temperatures.

Figure 15 illustrates the temperature-dependent characteristics of the relative dielectric constant of the insulation material. The abscissa is temperature, the ordinate is the relative dielectric constant of the insulation spacer material, the red polyline represents the average value, and the blue scatter points are the measured values. The relative dielectric constant shows a continuous upward trend with increasing temperature, and the measured values distribute around the average value, indicating that the relative dielectric constant of the insulation material has a significant temperature dependence; when the temperature rises from 25 °C to 120 °C, the average relative dielectric constant gradually increases from approximately 8 to 11.2, with an increase of 0.9 in the temperature range of 60~80 °C, demonstrating that the relative dielectric constant is more sensitive to temperature changes in this range; additionally, the deviation between the measured values and the average value is small, indicating that the data has good repeatability and stability.

## 3. Consider the Calculation and Analysis of the Mechanical Characteristics of the Windings Under the Temperatures of Each Component of the Transformer

**Figure 16:**
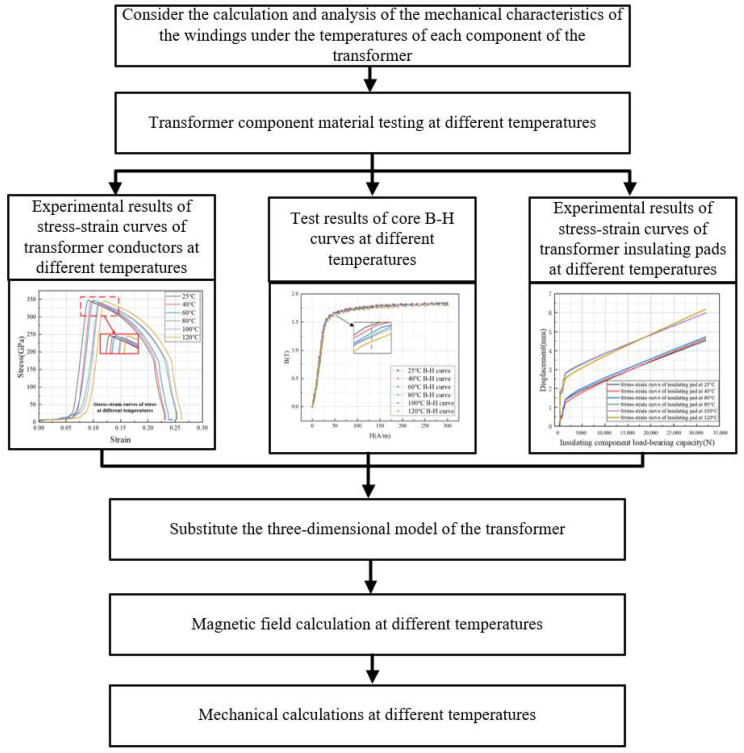
Calculation process of the mechanical properties of transformer windings considering the influence of thermal accumulation.

Figure 16 shows, this is the calculation and analysis of the mechanical properties of transformer windings considering the influence of thermal accumulation. First, we substitute the stress—strain curves of transformer winding conductors at different temperatures and the elastic modulus curves of transformer insulating components at different temperatures into the 3D model of the 110 kV transformer to calculate the mechanical properties of transformer windings, considering thermal accumulation.

Based on a three-phase three-limb transformer with a capacity of 50,000 kVA and a rated voltage of 110 kV, this study used finite element software to model and simulate the main transformer’s windings, iron core, and spacers. The specific model and parameters of the transformer are presented in Table 2.

### 3.1. Model Establishment and Incentive Input

(1) Model Establishment

**Figure 17:**
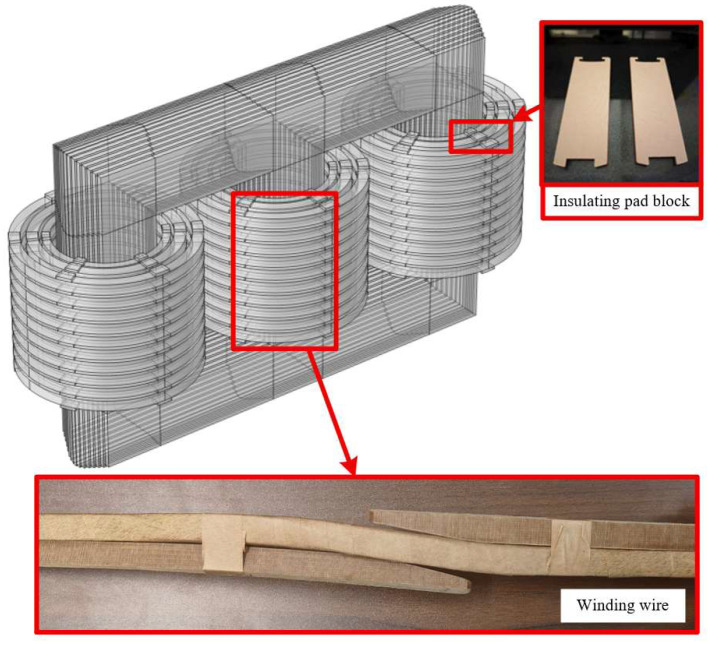
Three-dimensional simulation model of 110 kV transformer.

Based on Table 1, a 3D model of the 110 kV three-phase three-limb transformer was established. To balance computational efficiency and model rationality, reasonable simplifications of some structural and process factors are required. The influence of mesoscopic process characteristics, such as the coil winding method and transposition process, on the magnetic field. Omit structural components, such as end rings, pressure plates, and lead wires, which have a weak influence on the overall magnetic field distribution. Through such simplifications, on the premise of retaining the core electromagnetic–structural characteristics of the transformer, the model complexity can be effectively reduced, and the efficiency of the numerical solution can be improved. The 3D model of the 110 kV three-phase three-limb transformer is shown in Figure 17.

After establishing the model of the 110 kV three-phase three-limb transformer, comprehensively considering the material properties such as the magnetization curve (B-H curve) of the core, the stress–strain constitutive relation of the windings at different temperatures, as well as the elastic modulus of the insulation components at different temperatures and the numerical simulation calculation of the leakage magnetic field, was and calculated. The results obtained from the leakage magnetic field simulation were coupled to the structural mechanics field, and the mechanical response characteristics of the transformer at different temperatures were solved.

(2) Excitation Input

**Figure 18:**
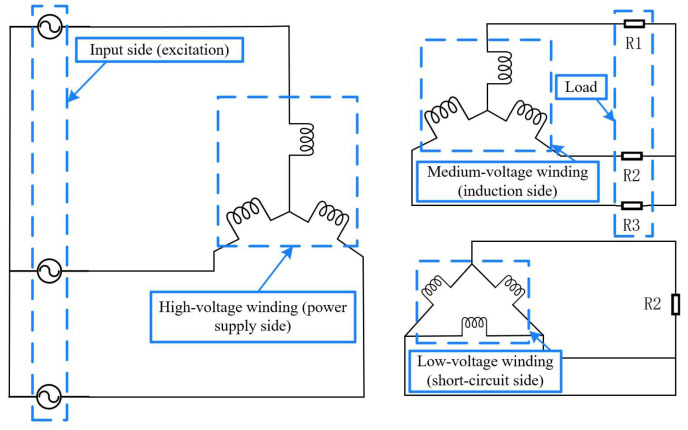
Wiring diagram of low-voltage two-phase short-circuit for 110 kV power transformer.

**Figure 19:**
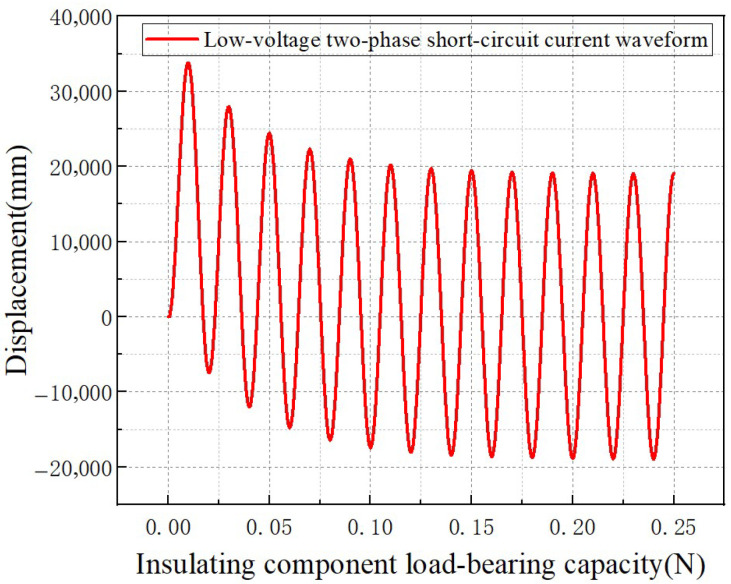
Low-voltage two-phase short-circuit current waveform.

This paper adopts the low-voltage two-phase short-circuit condition of a 110 kV power transformer, and selects the time of the short-circuit current at the maximum amplitude for multi-physics field comparison under varying temperatures. The wiring diagram of the low-voltage two-phase short-circuit of the 110 kV power transformer is shown in Figure 18. The input short-circuit current excitation is shown in Figure 19.

### 3.2. Calculation Results of Transformer Magnetic Field at Different Temperatures

(1) Theory of Electromagnetic Calculation for Power Transformers under Varying Temperatures

Maxwell’s equations describe the macroscopic properties of electromagnetic fields and accurately reflect the interrelationships between various physical quantities in the internal electromagnetic fields of electrical equipment such as transformers. In finite element calculations, equipment like transformers is regarded as a quasi-static magnetic system, and the influence of displacement current can be neglected (∂D/∂t=0); therefore, the differential form of Maxwell’s field equations for magnetic fields can be expressed as

(5) ∇×H=J

(6) $$\nabla \times E=-\frac{\partial B}{\partial t}$$

(7) ∇⋅B=0

where B, E, J are, respectively, the magnetic field strength (A/m), magnetic induction intensity, which is also known as magnetic flux density (T), magnetic induction intensity (V/m), and current density (A/m^2^).

The constitutive equations describe the relationships between the field vectors: magnetic field strength H, magnetic induction intensity B, magnetic induction intensity E, and current density J are, respectively,

(8) B=μH

(9) J=σE

where μ and σ are, respectively, permeability and conductivity.

Based on the definition of the magnetic vector potential A and Equation (6), the relationship between the magnetic vector potential A, scalar electric potential ϕ, magnetic flux density B, and electric field strength E can be obtained as

(10) B=∇×A

(11) $$E=-\frac{\partial A}{\partial t}-\nabla \Phi$$

**Figure 20:**
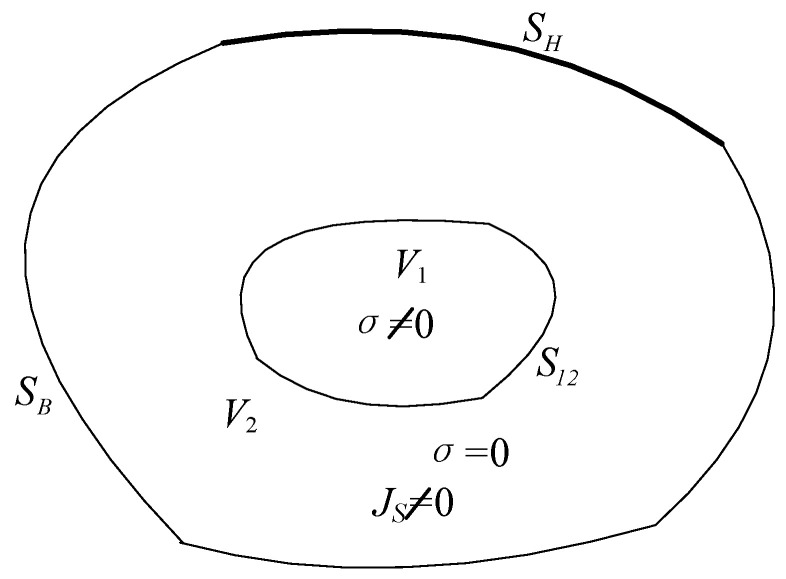
Layout of eddy current problem solving.

Figure 20 shows the typical solution domain V for eddy current-containing problems in the numerical calculation of electromagnetic fields of electrical equipment such as transformers. Here, $V_{1}$ is the eddy current region, which contains conductive media (e.g., transformer core) and no source currents; $V_{2}$ is the non-eddy current region, which includes given source currents (e.g., transformer windings). By substituting Equations (10) and (11) into Equations (5) and (6), and combining with Equations (8) and (9), the field equations of the (A,ϕ−A) method for solving three-dimensional eddy current problems in regions $V_{1}$ and $V_{2}$ can be derived.

(12) $$V_{1}:\left\{\begin{array}{c} \nabla \times (\nu \nabla \times A)-\nabla (\nu \nabla \cdot A)+\sigma \frac{\partial A}{\partial t}+\sigma \nabla \phi =0\\ \nabla \cdot \left(-\sigma \frac{\partial A}{\partial t}-\sigma \nabla \phi \right)=0 \end{array}\right.$$

(13) $$V_{2}:\nabla \times \left(\nu \nabla \times A\right)-\nabla \left(\nu \nabla \cdot A\right)=J_{S}$$

where v is magnetoresistivity, which is equal to 1/μ $J_{s}$ is the source current density (A/m^2^).

Within the solution domain V, the boundary conditions and interface conditions satisfied by A and ϕ are

(14) $$S_{B}:\left\{\begin{array}{l} n\times A=0\\ v\nabla \cdot A=0 \end{array}\right.$$

(15) $$S_{H}:\left\{\begin{array}{c} n\cdot A=0\\ (\nu \nabla \times A)\times n=0 \end{array}\right.$$

(16) $$S_{12}:\left\{\begin{array}{c} A_{1}=A_{2}\\ v_{1}\nabla \cdot A_{1}=v_{2}\nabla \cdot A_{2}\\ v_{1}\nabla \times A_{1}\times n_{12}=v_{2}\nabla \times A_{2}\times n_{12}\\ n\cdot \left(-\sigma \frac{\partial A}{\partial t}-\sigma \nabla \phi \right)=0 \end{array}\right.$$

To ensure the uniqueness of the solution, the Coulomb gauge is adopted, which specifies that the divergence of A is 0.

(17) ∇⋅A=0

For the core cross-section satisfying the parallel boundary condition shown in Equation (15), all element nodes on the surface are set to

(18) $$\begin{array}{l} A_{x}=0\\ A_{y}=0\\ \frac{\partial A_{z}}{\partial z}=0 \end{array}$$

For other interfaces between the transformer and air, they satisfy the boundary condition given in Equation (18), which is a natural interface condition and is automatically satisfied during the finite element calculation. The selected solution domain is considered sufficiently large, and the electromagnetic field on the five air boundaries has approximately decayed to zero; therefore, all nodes are set to infinite boundary conditions.

(19) $$\left\{\begin{array}{l} A_{x}=0\\ A_{y}=0\\ A_{z}=0\\ \Phi =0 \end{array}\right.$$

The model transformer is composed of curved silicon steel sheets, and its permeability μ exhibits significant nonlinearity with the change in magnetic field strength. Therefore, it is necessary to assign nonlinear magnetic characteristics to the core solution domain. To achieve higher solution accuracy, relevant experiments on the actual model transformer are designed, and the B−H curve of the transformer during actual operation is calculated through the primary side current and secondary side voltage. Load it into the material properties of the transformer core to complete the assignment of the material properties of the silicon steel sheets.

In the finite element analysis and calculation of electromagnetic fields, the coupling between the circuit and the magnetic field is achieved through the loading of the field source current density in the non-eddy current region. The field equations of the (A,ϕ−A) method in the eddy current region and non-eddy current region are shown in Equations (12) and (13), respectively. For the analysis model shown in Figure 20, the core solution domain is the eddy current region, and other solution domains, such as coils and air, are non-eddy current regions. Moreover, the coils are electromagnetic field source current regions; that is,$J_{s}$ is no longer zero, and its results are derived from the circuit calculation results in the previous section. In polar coordinates, the current density in the coils is considered to be uniformly distributed; therefore, during the finite element calculation, $J_{s}$ is consistent for all element nodes in the coil solution domain. Thus, the coupled analysis and calculation of the circuit and the magnetic field are realized.

(2) Calculation Results and Analysis

**Figure 21:**
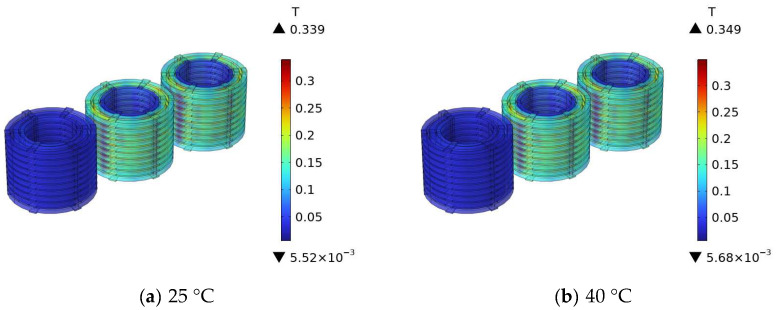

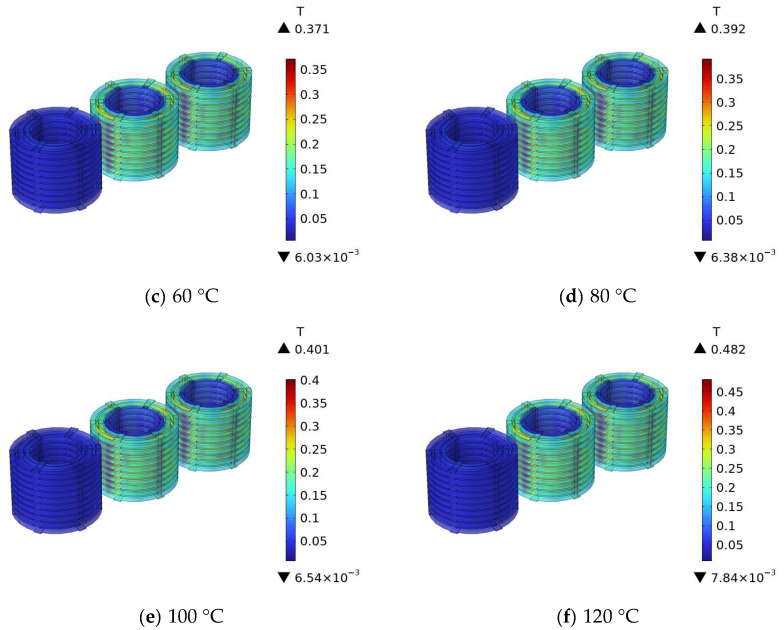
Contour map of magnetic field simulation calculation for 110 kV transformer at different operating temperatures.

Figure 21 shows the simulation results of the winding magnetic field of the 110 kV transformer at different temperatures.

Figure 21a–f show the simulation contour maps of the magnetic field distribution in the transformer windings at different operating temperatures. As the operating temperature gradually increases from 25 °C to 120 °C, the magnetic field intensity in the transformer winding area shows an obvious increasing trend: the maximum magnetic field intensity is approximately 0.339 T at 25 °C, increases to 0.349 T at 40 °C, reaches 0.371 T at 60 °C, further increases to 0.392 T at 80 °C, is 0.401 T at 100 °C, and climbs to 0.482 T at 120 °C. This indicates that temperature is an important factor affecting the magnetic field distribution of transformer windings, and an increase in temperature causes the intensity of the magnetic field where the windings are located to increase significantly, reflecting a positive correlation between temperature and winding magnetic field intensity.

As can be seen from the simulation results, with the increase in temperature, the winding magnetic field increases. The main reason for this phenomenon is that as temperature rises, the magnetic permeability of the transformer core decreases, the main magnetic flux reduces, which in turn leads to an increase in the magnetic field strength in the winding region [26].

### 3.3. Mechanical Calculation Results of Transformer at Different Temperatures

#### 3.3.1. Simulation Calculation of Axial Electromagnetic Force for 110 kV Transformer at Different Temperatures

(1) The Calculation Theory of Electromagnetic Force for Power Transformers

Since the electrodynamic force generated by the short-circuit current is related to its instantaneous value, it is necessary to determine the maximum instantaneous value of the short-circuit current. This maximum instantaneous value occurs approximately half a cycle after the short circuit; when f = 50 Hz, this time should be 0.01 s. This maximum instantaneous value of the short-circuit current is also referred to as the impact short-circuit current, ich, and its value is as follows:

(20) $$i_{\mathrm{ch}}=\frac{U_{m}}{Z}\sin(\pi -\frac{\pi }{2})+\frac{U_{m}}{Z}e^{-\frac{R}{L}0.01}=\frac{U_{m}}{Z}(1+e^{-\frac{R}{L}0.01})=\frac{U_{m}}{Z}K_{\mathrm{ch}}$$

Among them, K*_ch_*= 1 + e ^(−R × 0.01/L)^ is the impact coefficient of the short-circuit current, √2 K*_ch_* is the impact coefficient of the asymmetric short-circuit current, X is the sum of the transformer reactance X_t_ and the system reactance X_s_, R is the sum of the transformer resistance R_t_ and the system resistance R_s_, and U_m_ is the amplitude of the power supply voltage.

**Figure 22:**
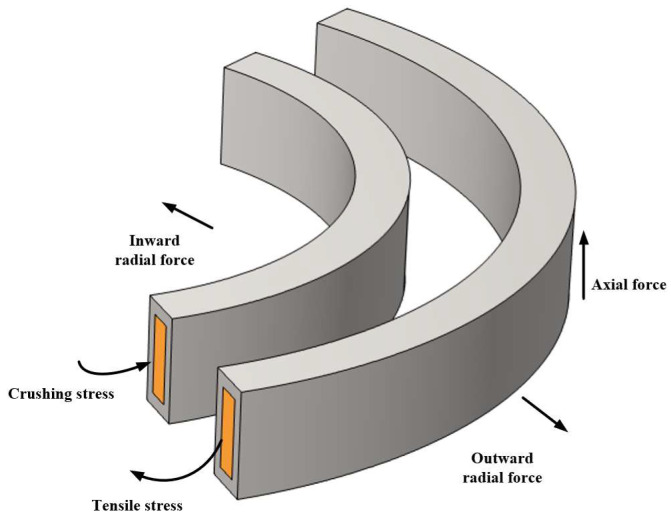
Schematic diagram of forces acting on transformer windings.

The current in the winding generates axial leakage fluxes B_x_ and B_y_ in the axial direction, which interact with the current in the winding to produce a radial force F_x_. The radial force causes the outer winding to expand radially outward and the inner winding to compress radially inward; therefore, the radial force ultimately increases the insulation distance of the main air gap. A schematic diagram of the axial force and radial force acting on the transformer winding is shown in Figure 22.

According to the Lorentz force formula, the radial force per unit length of the winding disk can be obtained:

(21) $$F_{x}=B_{y\max}i_{\mathrm{ch}}$$

Among them, B*_ymax_* is the maximum axial magnetic flux density under sudden short-circuit conditions, and ich is the maximum value of the short-circuit current under sudden short-circuit conditions.

According to the Lorentz force formula, the axial force per unit length of the winding disk can be obtained:

(22) $$F_{y}=B_{x\max}i_{\mathrm{ch}}$$

Among them, B*_xmax_* is the maximum radial magnetic flux density under sudden short-circuit conditions. ich is the maximum value of the short-circuit current under sudden short-circuit conditions.

(2) Calculation Results and Analysis

**Figure 23:**
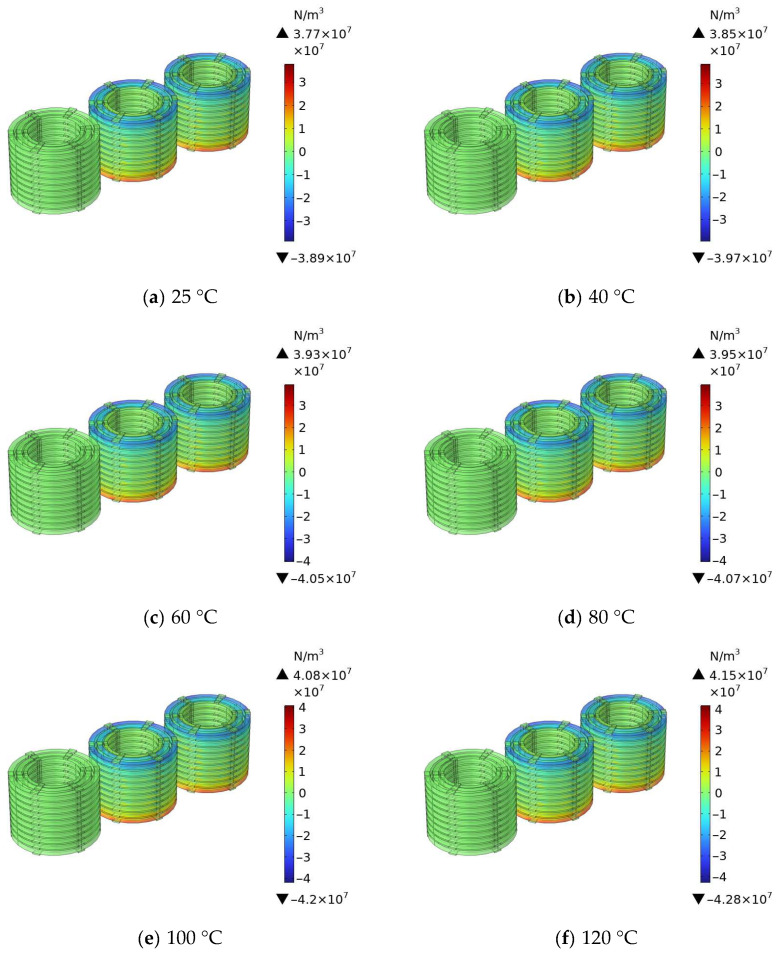
Contour map of simulation calculation for axial electromagnetic force of 110 kV transformer at different operating temperatures.

After calculating the magnetic field in the transformer windings at different temperatures, the axial electromagnetic force of the windings at different temperatures was calculated to verify the influence of temperature on the axial electromagnetic force of the transformer. The simulation results are shown in Figure 23.

Figure 23 shows the simulation contour map of the winding axial electromagnetic force for the 110 kV transformer at different operating temperatures. It can be seen from the figure that as the operating temperature gradually increases from 25 °C to 120 °C; the axial electromagnetic force of the transformer windings shows an obvious increasing law: the maximum axial electromagnetic force is 3.77 × 10^7^ N/m^3^ at 25 °C, increases to approximately 3.85 × 10^7^ N/m^3^ at 40 °C, reaches 3.93 × 10^7^ N/m^3^ at 60 °C, further rises to 3.95 × 10^7^ N/m^3^ at 80 °C, is 4.08 × 10^7^ N/m^3^ at 100 °C, and climbs to 4.15 × 10^7^ N/m^3^ at 120 °C. This indicates that the operating temperature has an important influence on the axial electromagnetic force of the transformer windings, and an increase in temperature causes the axial electromagnetic force exerted on the windings to increase significantly.

#### 3.3.2. Simulation Calculation of Radial Electromagnetic Force for 110 kV Transformer at Different Temperatures

**Figure 24:**
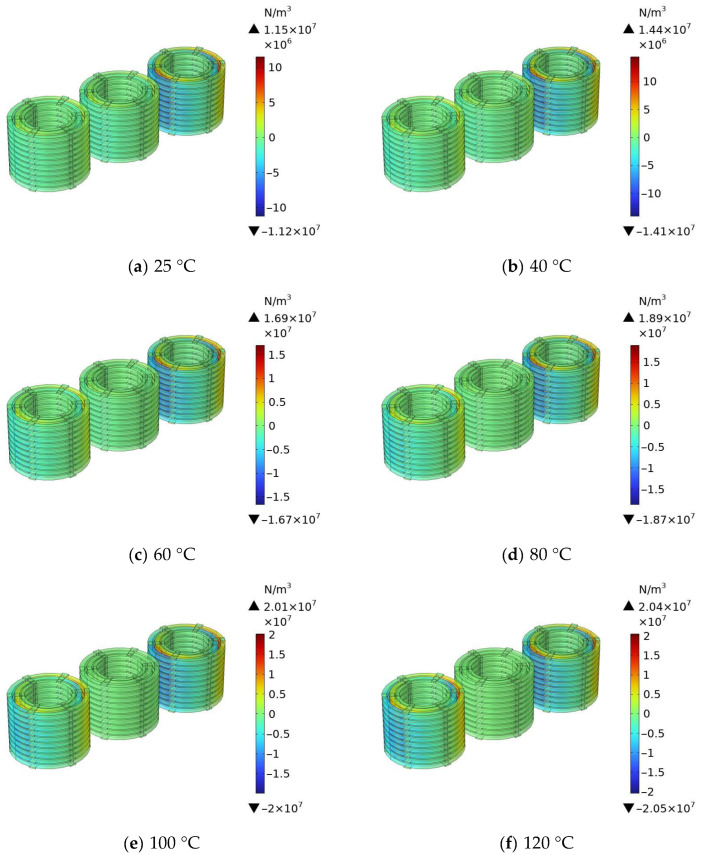
Contour map of simulation calculation for radial electromagnetic force of 110 kV transformer at different operating temperatures.

After completing the calculation of the magnetic field in the windings of the 110 kV transformer at different temperatures, the simulation calculation of the radial electromagnetic force of the windings at different temperatures was carried out to verify the influence of temperature on the radial electromagnetic force. The simulation results are shown in Figure 24.

The simulation analysis of the radial electromagnetic force of transformer windings at 25 °C to 120 °C shows that the radial electromagnetic force increases significantly with an increase in temperature. Its maximum value is 1.15 × 10^7^ N/m^3^ at 25 °C and reaches 2.04 × 10^7^ N/m^3^ at 120 °C, with the amplitude being nearly twice that of the initial temperature; at different temperatures, the electromagnetic force concentrates at the winding ends, and the rise in temperature aggravates the concentration effect. At low temperatures, there are only a few high electromagnetic force areas at the outer ends; at high temperatures, these high electromagnetic force areas expand inward and the gradient increases, and at high temperatures, the winding electromagnetic force has a larger amplitude and a more prominent end concentration effect, which easily causes insulation wear or winding deformation.

#### 3.3.3. Simulation Calculation of Winding Displacement for 110 kV Transformer at Different Temperatures

(1) The Calculation Theory of Winding Displacement for Power Transformers

**Figure 25:**
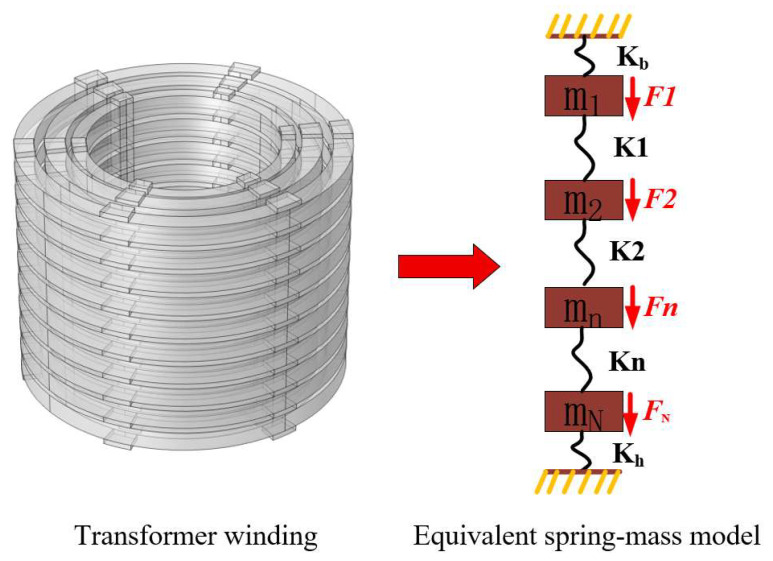
Axial mass—spring model of transformer windings.

The winding disks are equivalent to mass elements, and the end insulation of the windings and the spacers between the winding disks are equivalent to springs. A “mass-spring system” model for the axial vibration of transformer windings is established, as shown in Figure 25.

The motion equation of each mass element is as follows:

(23) $$\left\{\begin{array}{l} \;\;\;m_{1}\frac{d^{2}z_{1}}{dt^{2}}+c_{1}\frac{dz_{1}}{dt}+k_{B}z_{1}+k_{1}(z_{1}-z_{2})=F_{1}+m_{1}g\\ m_{2}\frac{d^{2}z_{2}}{dt^{2}}+c_{2}\frac{dz_{2}}{dt}-k_{1}(z_{1}-z_{2})+k_{2}(z_{2}-z_{3})=F_{2}+m_{2}g\\ \ldots \ldots \ldots \ldots \ldots \ldots \ldots \ldots \ldots \ldots \ldots \ldots \ldots \ldots \ldots \ldots \ldots \ldots \ldots \ldots \ldots \ldots \\ m_{n}\frac{d^{2}z_{n}}{dt^{2}}+c_{n}\frac{dz_{n}}{dt}-k_{n-1}(z_{n-1}-z_{n})+k_{n}(z_{n}-z_{n+1})=F_{n}+m_{n}g\\ \ldots \ldots \ldots \ldots \ldots \ldots \ldots \ldots \ldots \ldots \ldots \ldots \ldots \ldots \ldots \ldots \ldots \ldots \ldots \ldots \ldots \ldots \ldots \\ m_{N}\frac{d^{2}z_{N}}{dt^{2}}+c_{N}\frac{dz_{N}}{dt}-k_{N-1}(z_{N-1}-z_{N})+k_{H}z_{N}=F_{N}+m_{N}g \end{array}\right.$$

Among them, m_n_ is the mass of unit n, k_n_ is the elastic coefficient of the spacer between winding disk n and winding disk n + 1, k_B_ and k_h_ are the elastic coefficients of the insulation spacers at the winding ends, z_N_ is the displacement of the nth unit relative to its original position, cn is the friction coefficient, m_N_d^2^z_N_/dt^2^ is the inertial force of the nth mass element, c_N_dz_N_/dt is the friction force of the nth mass element in oil or air, k_B_z_1_, k_n−1_(z_n−1_ − z_n_), k_n_(z_n_ − z_n+1_), and k_H_z_N_ are elastic forces, F_N_ is the electromagnetic force acting on the nth unit, and m_n_g is the weight of the nth unit. When the transformer is in operation, the temperatures of the upper and lower parts inside are different, resulting in different elastic coefficients of the transformer insulation spacers. In the spring–mass model, the influence of temperature on the axial displacement of the transformer winding is reflected through parameters such as k_B_, k_H_, and k_1_.

(2) Calculation Results and Analysis

**Figure 26:**
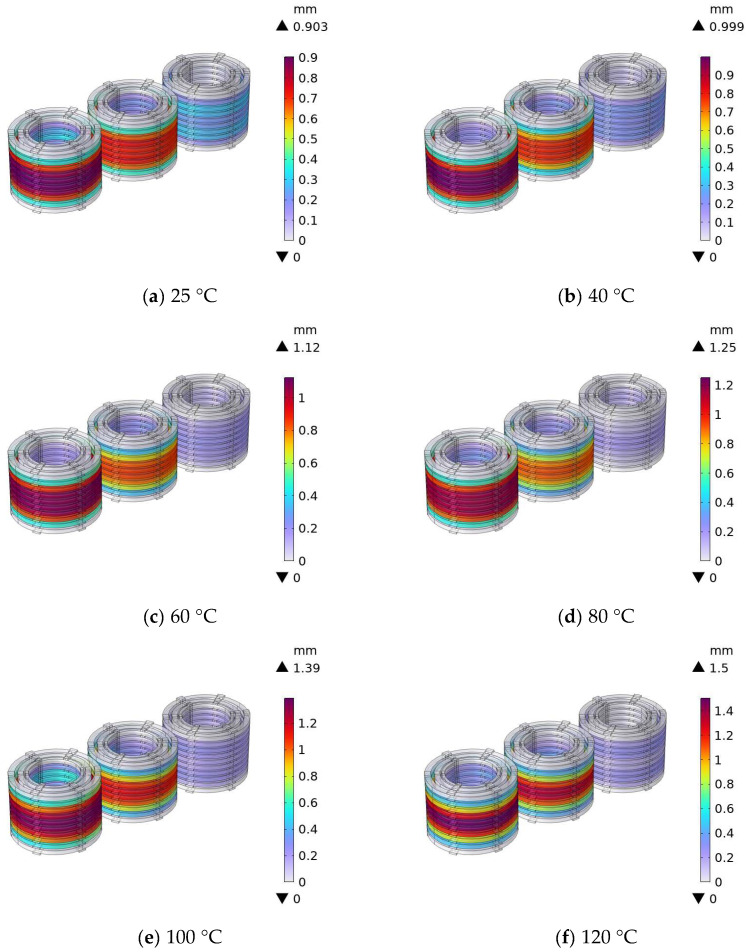
Contour map of simulation calculation for winding displacement of 110 kV transformer at different operating temperatures.

The simulation calculation results of the winding displacement for the 110 kV transformer at different temperatures are shown in Figure 26.

From the simulation results of the winding displacement of the 110 kV transformer at different temperatures, it can be seen that the winding displacement increases significantly with increasing temperature: the maximum displacement is approximately 0.903 mm at 25 °C, increases to approximately 1.5 mm at 120 °C, and the displacement is concentrated in the winding end area. The higher the temperature, the wider the range of the displacement area at the end, and the more severe the degree of displacement. This indicates that high temperatures aggravate the displacement deformation of the windings, and long-term operation may affect the insulation and structural stability of the windings.

## 4. Prediction of Transformer Winding Short-Circuit Displacement Based on Random Forest

In the prediction of transformer winding short-circuit displacement, the Random Forest, through the ensemble learning mechanism of multiple decision trees, has a strong anti-overfitting ability. Even if there is minor noise or local anomalies in the training data, it can reduce the bias of a single tree by voting among multiple trees, thereby ensuring high-precision prediction on the test set. It can efficiently capture the impact of temperature and the number of impacts on the displacement.

### 4.1. Basic Introduction to Random Forest

Random Forest (RF) is an efficient machine learning model based on ensemble learning that improves the prediction stability and generalization performance by combining multiple decision trees. The core mechanism integrates the dual randomness of bagging and random feature selection. Sample Randomness: Bootstrap Sampling is adopted to generate differentiated training subsets for each tree, and the unselected samples (out-of-bag (OOB) data) are used for unbiased estimation of model performance. Feature Randomness: Each tree selects features only from a random subset when splitting nodes, which reduces inter-tree correlation and enhances model diversity. A single decision tree performs recursive splitting based on the Gini index for decision classification or the mean squared error (MSE) for regression prediction, generating weak learners with high variance and low bias; the Random Forest leverages “collective wisdom” to balance bias and variance through the integrated results of majority voting (for classification) or mean aggregation (for regression). This improves the overall robustness by reducing the inter-tree correlation. The mathematical advantage of Random Forest lies in the significant improvement of model generalization performance and robustness through the organic combination of dual randomness and ensemble strategy.

From the original training set D (with a sample size of N), K different sub-training sets D_1_, D_2_, …, Dₖ are generated via random sampling with replacement. For each sub-training set Dₖ, when constructing a decision tree Tₖ, m features are randomly selected from all M features before splitting each node. The optimal splitting threshold is determined based only on these m features (to minimize the mean squared error (MSE) of the samples within the node), and the decision tree is not pruned, retaining its complete structure to ensure the diversity of individual trees.

For a certain node S, its mean squared error (MSE) is

(24) $$\mathrm{MSE}(S)=\frac{1}{\left|S\right|}\sum_{(x,y)\in S}{\left(y-{\bar{y}}_{S}\right)}^{2}$$

This, |S| represents the number of samples contained in node S, ${\bar{y}}_{S}$ represents the mean value of the target values of the samples within node S.

(25) $${\bar{y}}_{S}=\frac{1}{\left|S\right|}\sum_{(x,y)\in S}y$$

When node S is split into the left child node SL and right child node S_R_, and is based on the threshold t of feature j, the total MSE after splitting is

(26) $$\mathrm{MSE}(S_{L},S_{R})=\frac{\left|S_{L}\right|}{\left|S\right|}\mathrm{MSE}(S_{L})+\frac{\left|S_{R}\right|}{\left|S\right|}\mathrm{MSE}(S_{R})$$

The algorithm iterates through m randomly selected features and all possible thresholds of each feature, and selects the feature–threshold combination that minimizes the total MSE to complete node splitting.

For the new sample x*, let the predicted value of the k-th decision tree Tₖ for it be Tₖ(x*), and then, the final predicted value of the Random Forest is the mean value of the predicted values of the K trees:

(27) $${\hat{y}}^{*}=\frac{1}{K}\sum_{k=1}^{K}T_{k}(x^{*})$$

Its OOB error for the regression task is

(28) $$\mathrm{OOB}\;\mathrm{Error}=\frac{1}{N}\sum_{i=1}^{N}(y_{i}-{\hat{y}}_{\mathrm{OOB}}(x_{i}){)}^{2}$$

where ${\hat{y}}_{\mathrm{OOB}}(x_{i})$ denotes the decision tree constructed using only the sub-training set that does not contain the i-th sample.

**Figure 27:**
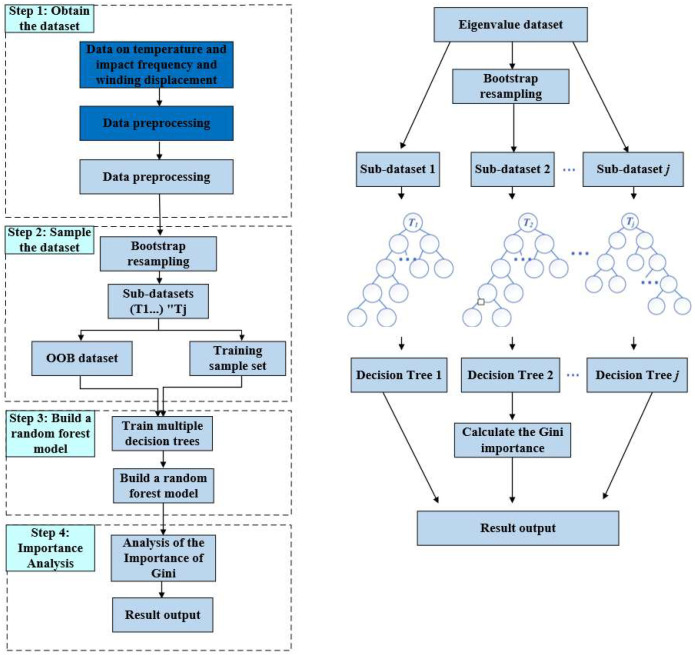
The flowchart and structure diagram of the RANDOM FOREST model.

Random Forest can quantify the contribution of each feature to the prediction results, which is commonly measured by the mean squared error reduction in regression tasks. For feature j, its importance Ij is the average value of the MSE reduction before and after node splitting when it participates in splitting across all decision trees:

(29) $$I_{j}=\frac{1}{K}\sum_{k=1}^{K}\left[\mathrm{MSE}(S_{k,j})-\left(\frac{\left|S_{k,j,L}\right|}{\left|S_{k,j}\right|}\mathrm{MSE}(S_{k,j,L})+\frac{\left|S_{k,j,R}\right|}{\left|S_{k,j}\right|}\mathrm{MSE}(S_{k,j,R})\right)\right]$$

where S_k,j_ denotes the node where feature j participates in splitting in the k-th tree, and S_k,j_, L and S_k,j,R_ denote the left and right child nodes of this node after splitting. The flowchart and structure of the Random Forest model are shown in Figure 27.

The prediction of the winding displacement of transformers under different temperatures and different numbers of impacts was based on the RF algorithm. First, the dataset was preprocessed, and feature dataset extraction was performed. Second, a sample division was conducted for the test and training sets. Finally, multiple decision trees were trained, and a Random Forest model was constructed.

### 4.2. Model Prediction and Analysis

**Figure 28:**
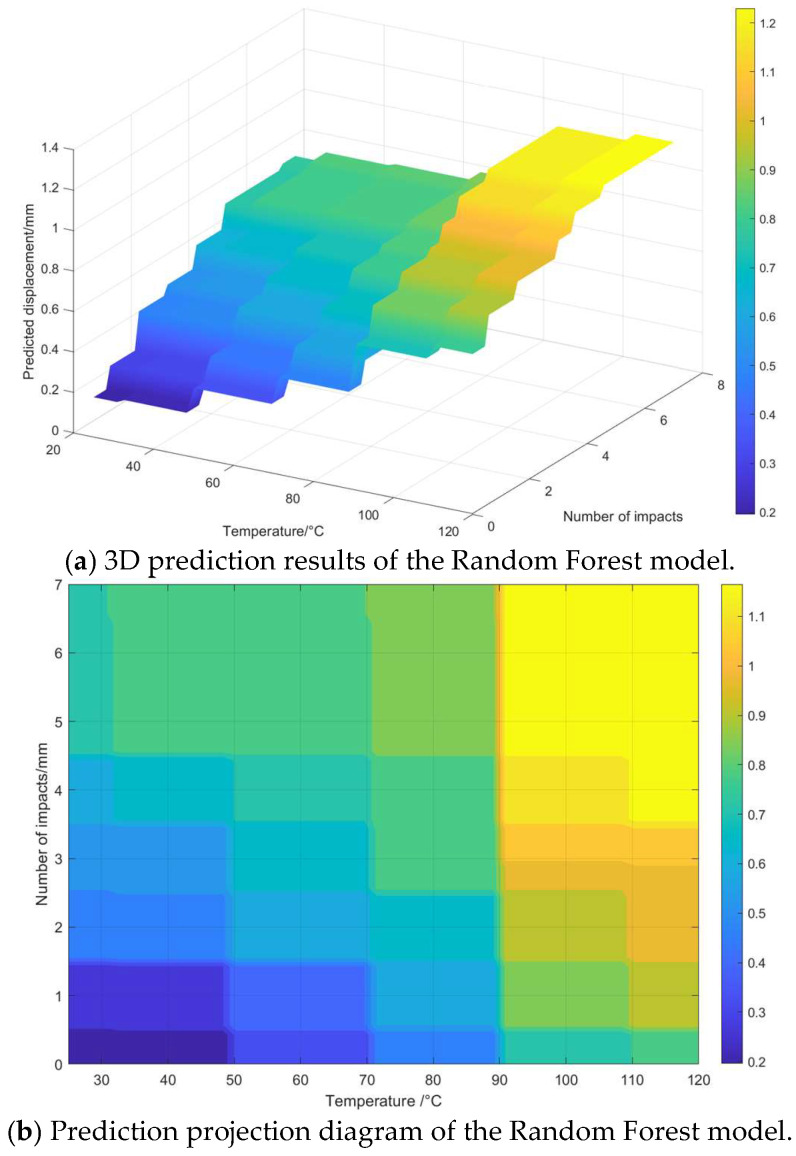
Prediction result diagrams of the Random Forest model.

Figure 28 shows the prediction result diagram of the Random Forest model for transformer winding displacement, where the horizontal axis represents the transformer operating temperature, the vertical axis represents the number of short-circuit impacts, and the *z*-axis represents the predicted winding displacement. As can be seen from Figure 28, the predicted winding displacement shows an obvious upward trend with an increase in the transformer operating temperature and the number of short-circuit impacts. When the operating temperature is relatively low and the number of short-circuit impacts is relatively small, the predicted value of the winding displacement is relatively low. When the operating temperature is relatively high and the number of short-circuit impacts is relatively large, the predicted value of the winding displacement increases significantly. This indicates that the operating temperature and the number of short-circuit impacts are key factors affecting the transformer winding displacement, and an increase in both will make the winding displacement more significant.

**Figure 29:**
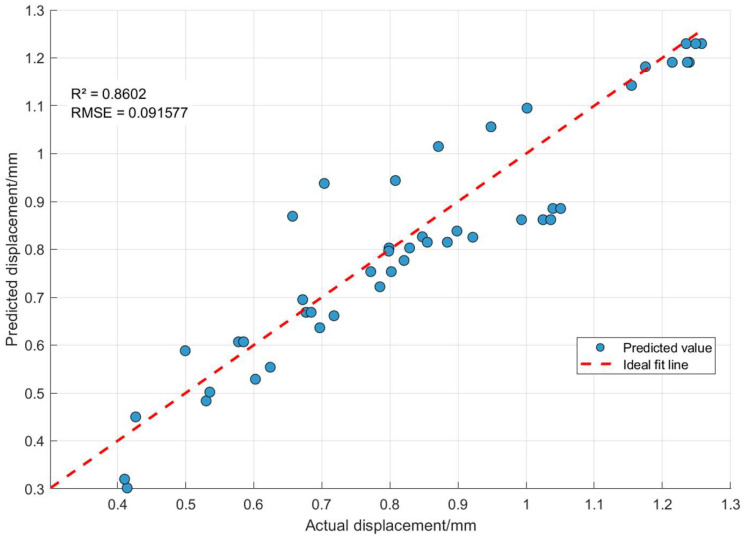
Comparison diagram of model prediction and test set results.

Figure 29 shows the fitting comparison diagram of the actual transformer winding displacement and the displacement predicted by the Random Forest model, where the horizontal axis represents the actual displacement and the vertical axis represents the predicted displacement; the blue scatter points are the predicted values of the model, and the red dashed line is the ideal fitting line where the predicted values completely coincide with the actual values. The mean squared error (MSE) between the predicted values and the actual values of the Random Forest model is 0.069501, the root mean squared error (RMSE) is 0.091577, and the coefficient of determination (R^2^) is 0.8602. From these error indicators, it can be concluded that the prediction error of the model is small and the overall prediction performance is excellent. From the analysis of the model error indicators, the coefficient of determination R^2^ was 0.9837, indicating an extremely strong linear correlation between the predicted displacement and the actual displacement, and the root mean squared error (RMSE) is 0.031280, reflecting a small average deviation between the predicted and actual values. Overall, the blue scatter points are closely distributed around the ideal fitting line, which indicates that the Random Forest model has high prediction accuracy and low error level for transformer winding displacement, and can accurately depict the variation law of the actual displacement.

**Figure 30:**
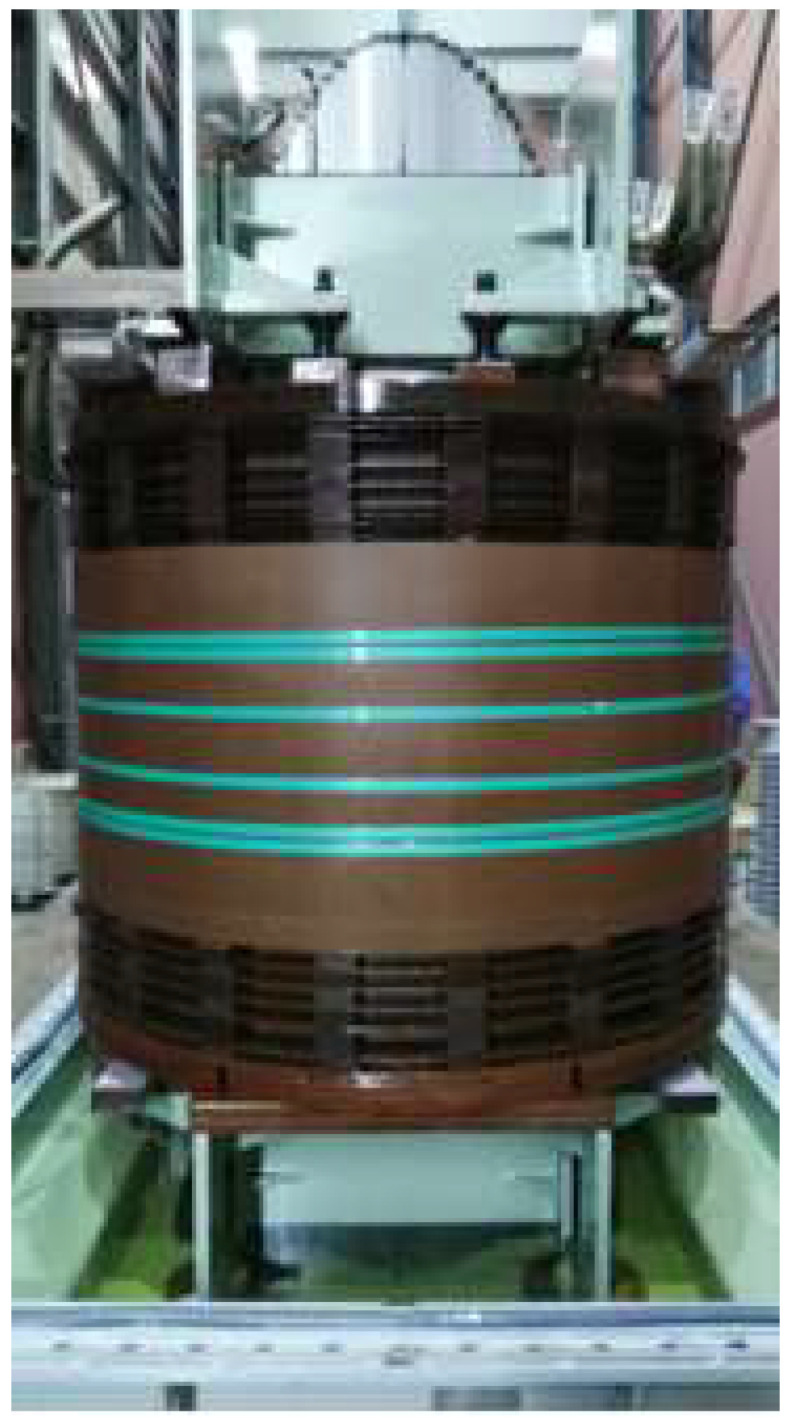
110 kV transformer for short-circuit test.

A factory acceptance test was conducted on a 110 kV transformer to verify that its windings and support structure would not suffer permanent deformation, displacement, or damage under the huge electrodynamic forces generated by a sudden short-circuit current. Figure 30 shows the transformer configured for the factory short-circuit test.

**Figure 31:**
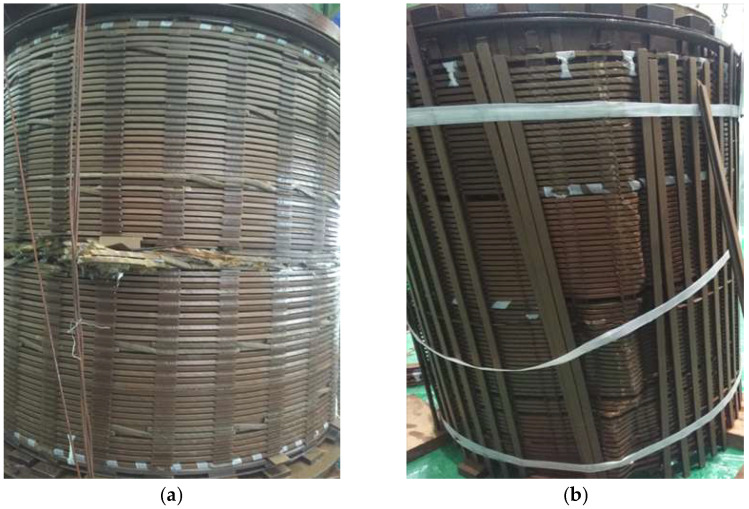
110 kV transformer hoisting and disassembly [25]. (**a**) Disassembly diagram of the high-voltage winding of the 110 kV transformer; (**b**) Disassembly drawing of the low-voltage winding of the 110 kV transformer.

As shown in Figure 31, the core inspection results after 15 short-circuit impacts on the 110 kV transformer indicate significant differences in the mechanical integrity of its windings. A clear radial unstable deformation occurred in the middle of the high-voltage winding, while the inter-stator insulation pads broke, and the axial insulation support bars were fractured. The low-voltage winding showed overall axial displacement, and loose traces were observed on the pressure plate and tightening bolts, indicating the failure of the axial compression system.

### 4.3. Verification of the 750 kV Transformer Scheme Based on the Method Proposed in This Paper

Following the methodology proposed in this study, simulation analysis was conducted on a main transformer with a unit capacity of 700 MVA, rated voltage of 750 kV, and model designation ODFPS-700,000/750 kV. Finite element software was employed to develop simulation models of the transformer’s windings, core, spacers, and support sticks. The detailed specifications and parameters of this transformer are presented in Table 3.

Magnetomechanical calculations on the windings of the 750 kV main transformer were performed at varying temperatures, with the results presented in Table 4.

Magneto–thermal–mechanical calculations were performed on the 750 kV main transformer at varying temperatures. The results demonstrate a monotonically increasing trend in the winding magnetic field, axial electromagnetic forces, radial electromagnetic forces, and displacements with rising temperature during transformer operation. These findings validate the methodology proposed in this study.

## 5. Conclusions

This study focuses on the mechanical calculation of a winding short circuit under short-circuit conditions for a 110 kV power transformer, considering the influence of thermal accumulation factors. The research content is as follows:

(1) Mechanical property tests were conducted on transformer conductor samples at different temperatures. The results showed that in the elastic deformation stage, as the temperature increased, the elastic modulus of the conductor decreased, indicating that the ability of the material to resist elastic deformation weakened. In the yield and strengthening stage, the higher the temperature, the more significant the decrease in the yield strength and tensile strength of the conductor, suggesting that the load-bearing capacity of the material decreased significantly under high temperatures. In the necking and fracture stage, high temperatures weakened the plastic deformation ability of the conductor and made it more prone to brittle fracture, with a deterioration in plasticity.
(2) Dynamic and static mechanical property tests were conducted on transformer insulation blocks at varying temperatures. The results indicate that as the temperature of the insulating components increases, the displacement of the insulation blocks gradually enlarges, while both the elastic modulus and yield strength exhibit a declining trend with rising temperature. This indicates that temperature is the key factor that weakens the deformation resistance of transformer insulation pads. And the dielectric constant of the insulating pad material was tested at different temperatures to further verify the impact of temperature on the transformer.
(3) The magnetic properties of iron-core silicon steel sheets at different temperatures were tested. The test results showed that as the test temperature increased, the magnetic flux density B of the silicon steel sheets gradually decreased. This would affect the leakage flux of the 110 kV transformer, thereby influencing its mechanical performance.
(4) Through simulation calculations for a 110 kV transformer, the simulation results showed that an increase in temperature significantly increased the intensity of the magnetic field in the winding, reflecting a positive correlation between temperature and the magnetic field intensity of the winding. The temperature has a significant impact on the axial and radial electromagnetic forces of the transformer winding, and an increase in temperature causes the axial and radial electromagnetic forces on the winding to increase significantly. High temperatures would exacerbate the displacement deformation of the winding, and the long-term operation may affect the insulation and structural stability of the winding.
(5) Based on the Random Forest algorithm, the influence of the temperature and impact frequency on the winding displacement was predicted. The Random Forest model was verified to have a high prediction accuracy and low error level for the displacement of the transformer winding, and could accurately depict the changes in the actual displacement. This provides a new method for assessing the short-circuit capacity of the transformer. The feasibility of the calculation method proposed in this paper was verified through a 750 kV main transformer.

## Figures and Tables

**Table 1:** Main performance parameters of the testing system.

Major Parameter	Mechanical Property
**Test temperature range**	20 °C~300 °C
**Size of silicon steel sheet**	150 mm × 150 mm
**Magnetic field intensity**	1 A/m~10,000 A/m
**Magnetic induction intensity**	0.001 T~2 T
**Frequency domain**	1 Hz~5 kHz

**Table 2:** Electrical parameters of 110 kV three-phase three-limb transformer [25].

Product Model	110 kV Transformer		
**Rated Frequency/Hz**	50		
**Core Structure**	Three-phase three-limb		
**Widing Parameters**	H-v winding	M-v winding	L-v winding
**Rated Capacity/kVA**	50,000	50,000	50,000
**Rated Voltage/kV**	110	38.5	10.5
**Winding Inner Radius/mm**	557.5	430	363
**Winding Outer Radius/mm**	645	512.5	408
**Total Winding Height/mm**	995	995	995
**Total Winding Turns**	478	167	79
**Spacer Dimensions (Length × Width)/mm**	87.5 × 19.18	82.5 × 22.9	45 × 27.45

**Table 3:** Parameters of 750 kV transformer.

Product Model	ODFPS-700,000/750		
Rated frequency/Hz	50		
Core structure	Single-phase four-column		
Winding parameters	H-v winding	M-v winding	L-v winding
Rated capacity/kVA	700,000	700,000	233,000
Rated voltage/kV	765	345	63
Inner radius of the winding/mm	1096	829.5	642
Outer radius of the winding/mm	1298	1001	729.5
Total height of the winding/mm	1970.48	2000.17	2000.5
Total number of turns in the winding	716	588	186
The number of struts × the width/mm	48 × 20	48 × 20	36 × 20
The number of cushion blocks per layer × the width/mm	48 × 40	48 × 40	36 × 30

**Table 4:** Magnetomechanical calculation results for windings.

Temperature/°C	Winding Magnetic Flux Density/T	Axial Electromagnetic Force/N/m^3^	Radial Electromagnetic Force/N/m^3^	Displacement/mm
25	0.53	4.32961 × 10^7^	6.25153 × 10^7^	5.98
40	0.59	4.48034 × 10^7^	6.26353 × 10^7^	7.69
60	0.67	4.50356 × 10^7^	6.27338 × 10^7^	9.28
80	0.73	4.51067 × 10^7^	6.27492 × 10^7^	10.4
100	0.77	4.52042 × 10^7^	6.27575 × 10^7^	11.5
120	0.82	4.52273 × 10^7^	6.27818 × 10^7^	13.6

## Data Availability

The original contributions presented in this study are included in the article. Further inquiries can be directed to the corresponding author.

## References

[1] Miao Y. (2024). Research on Radial Stability of Power Transformer Windings Considering Cumulative Effects. Ph.D. Thesis.

[2] Wang J. (2023). Research on the Short-Circuit Resistance Capacity of Power Transformers Considering Thermal Accumulation Effects. Ph.D. Thesis.

[3] Li C.Y., Wang Z. (2019). Experimental study on the influence of mechanical properties degradation of insulating paper in converter transformers due to mechanical and thermal effects. Proc. Chin. Soc. Electr. Eng..

[4] Zhang F., Li X.G., Zhu X.Y., Zhuang Z., Shi Y.H., Ji S.C. (2022). Evaluation Method for Short-Circuit Resistance Capacity of Transformer Windings Considering the Degree of Thermal Aging. Proc. Chin. Soc. Electr. Eng..

[5] Bakshi A. (2015). An investigation of winding curvature effect on the mechanical strength of transformer windings. IEEE Trans. Power Deliv..

[6] Wang Z.X., Zhang S.Q., Xu Z.Y. (2022). Test and evaluation of the bending resistance of transformer self-adhesive reversal conductors under multiple factors conditions. High Volt. Eng..

[7] Sinha A., Kaur S. (2016). Analysis of short circuit electromagnetic forces in transformer with asymmetrically placed windings using Finite Element Method. Proceedings of the 2016 Second International Innovative Applications of Computational Intelligence on Power, Energy and Controls with their Impact on Humanity (CIPECH).

[8] Ahn H.M., Lee J.Y., Kim J.K., Oh Y.H., Jung S.Y., Hahn S.C. (2011). Finite-element analysis of short-circuit electromagnetic force in power transformer. IEEE Trans. Ind. Appl..

[9] Bakshi A. (2019). Effect of width of axial supporting spacers on the buckling strength of transformer inner winding. IEEE Trans. Power Deliv..

[10] Liu J.J. (2023). Analysis of leakage flux and winding short-circuit vibration in oil-immersed transformers. Transformer.

[11] Yadav S., Mehta R.K. FEM based study of short circuit forces on power transformer windings. Proceedings of the 2019 3rd International Conference on Recent Developments in Control, Automation & Power Engineering (RDCAPE).

[12] Luo H.W., Lai W.Q., Jiang G.Y., Lin N., Cui S.W., Jiang Y., Li M.Q. (2017). Study on elastic modulus of transformer winding materials and axial mechanical properties under short circuit at different temperatures. Insul. Mater..

[13] Muhamad N.A., Kamarden H., Othman N.A. (2015). Heat distribution pattern of oil-filled transformer at different hottest spot temperature locations. Proceedings of the 2015 IEEE 11th International Conference on the Properties and Applications of Dielectric Materials (ICPADM).

[14] Zhang B. (2018). Research on the Dynamic Thermal Stability of Power Transformers Based on Multi-Physical Field Coupling. Ph.D. Thesis.

[15] Aboura F., Touhami O. (2017). Effect of the GICs on magnetic saturation of asymmetric three-phase transformer. IET Electr. Power Appl..

[16] He B., Martens J., Zhang G., Botev A., Brock A., Smith S.L., Teh Y.W. (2023). Deep transformers without shortcuts: Modifying self-attention for faithful signal propagation. arXiv.

[17] Jing Y.T., Wang N., Li Y., Guo H. (2019). Research on temperature rise of transformer windings under electromagnetic-heat-flow weak coupling. J. Electr. Mach. Control..

[18] Li Z.X., Zheng Y.Y., Li G., Fu H.Q., Luo P.F., Hong Z.M. (2020). Fault analysis of a 220kV transformer with self-adhesive interleaved conductors used for the low-voltage winding. Transformer.

[19] Wang J. (2023). Research on the Anti-Surge Capacity of Power Transformers Considering Thermal Accumulation Effects. Ph.D. Thesis.

[20] Han J.B. (2025). Research on multi-fault diagnosis method for electrical equipment based on random forest. Electr. Equip. Econ..

[21] Cai L., Wang Z., Li J.Y., He W., Shi C.L. (2024). Research on intelligent detection technology for transformer faults based on improved random forest algorithm. Adhesion.

[22] Wang L., Chu M.Y., Wang X.H., Guang H.L., Chen P., Gao G.L. (2024). Research on operation status monitoring method for primary equipment in smart substation based on random forest. Electr. Meas. Instrum..

[23] Luo Y. (2023). Exploration of an early warning method for abnormal states of 220kV main transformers based on random forest. Guangxi Electr. Power.

[24] Luo L., Li Y., Shi Y., Han T., Yang W.C., Jing X.J. (2023). Research on transformer temperature monitoring and fault prediction method based on fog computing and random forest algorithm. Transformer.

[25] Zhou X., Ma Y., Tian T., Wang X., Jin H., Chen D., Xin Y., Wang S. (2025). Calculation and analysis of mechanical properties of transformer windings accounting for actual operating characteristics. AIP Adv..

[26] Li Y.J., Yan X.X., Zhang C.G., Chen Y.F., Ying W.L. (2020). Calculation of Transformer Loss and Prediction of Hot Spot Based on Magneto-Thermo-Fluid Coupling Model. Trans. China Electrotech. Soc..

